# Role of Traditional Chinese Medicine in the Management of Viral Pneumonia

**DOI:** 10.3389/fphar.2020.582322

**Published:** 2020-10-22

**Authors:** Shengyan Xi, Yunhong Li, Lifeng Yue, Yuewen Gong, Linchao Qian, Tengxiao Liang, Yong’an Ye

**Affiliations:** ^1^ Department of Traditional Chinese Medicine, School of Medicine, Xiamen University, Xiamen, China; ^2^ Department of Traditional Chinese Medicine, Xiang’an Hospital of Xiamen University, Xiamen, China; ^3^ The 3rd Neurology Department, Emergency Department, Gastroenterology Department, Dongzhimen Hospital, Beijing University of Chinese Medicine, Beijing, China; ^4^ College of Pharmacy, Rady Faculty of Health Sciences, University of Manitoba, Winnipeg, MB, Canada; ^5^ School of Traditional Chinese Medicine, Xiamen University Malaysia, Sepang, Malaysia

**Keywords:** traditional Chinese medicine, viral pneumonia, severe acute respiratory syndrome coronavirus, influenza virus, coronavirus induced disease 2019

## Abstract

Viral pneumonia is one kind of acute respiratory tract infection caused by the virus. There have been many outbreaks of viral pneumonia with high contagiousness and mortality both in China and abroad, such as the great influenza in 1918, the severe acute respiratory syndrome (SARS) coronavirus in 2003, the Influenza A (H1N1) virus in 2009, and the Middle East Respiratory Syndrome coronavirus (MERS-CoV) in 2012 and the severe acute respiratory syndrome coronavirus 2 (SARS-CoV-2) in 2019. These outbreaks and/or pandemic have significant impact on human life, social behaviors, and economic development. Moreover, no specific drug has been developed for these viruses. Traditional Chinese medicine (TCM) plays an important role in the treatment of viral pneumonia during these outbreaks especially in SARS and SARS-CoV-2 because studies suggest that TCM formulations may target several aspects of the disease and may have lesser side effects than manufactured pharmaceuticals. In recent years, a lot of clinicians and researchers have made a series of in-depth explorations and investigations on the treatment of viral pneumonia with TCM, which have understood TCM therapeutic mechanisms more specifically and clearly. But critical analysis of this research in addition to further studies are needed to assess the potential of TCM in the treatment of viral pneumonia.

## Introduction

Viral pneumonia is an acute respiratory infectious disease caused by viruses with different degrees of contagiousness. The main clinical manifestation is fever, which may be accompanied by symptoms such as anhidrosis or sweating, nasal congestion, runny nose, sore throat and cough (Figueiredo, 2009). Common viruses that cause pneumonia include adenovirus, coronavirus, human metapneumovirus, rhinovirus, respiratory syncytial virus, influenza virus and parainfluenza virus (Jain, 2017). Among them, severe acute respiratory syndrome (SARS) coronavirus (SARS-CoV) in 2003, Influenza A (H1N1) virus in 2009, and middle east respiratory syndrome coronavirus (MERS-CoV) in 2012 and severe acute respiratory syndrome coronavirus 2 (SARS-CoV-2) or called novel coronavirus in 2019 are highly contagious and fatal. As of 30 May 2020, there were 5,817,385 confirmed cases and 362,705 deaths in the coronavirus induced disease 2019 (COVID-19) outbreak since December 2019, and the trend is still on the rise (World Health Organization, 2020). At present, the commonly-used antiviral drugs in western medicine are probavirin, acyclovir, interferon, adenosine arabine, etc., which are easy to produce drug resistance, have many side effects and poor efficacy as well as other disadvantages (Amarelle et al., 2017). Because no specific and effective antiviral drugs have been developed in western medicine and Chinese herbal medicine possess clinical features of targeting multiple components and having multiple approaches, traditional Chinese medicine (TCM) has unique advantages in relieving symptoms, shortening treatment time and reducing the development of severe pneumonia. In the fight against COVID-19, the State Administration of Traditional Chinese Medicine of China has actively promoted the therapeutic role of TCM. As the member of the Leading Group of the National Health Commission and Secretary of the Leading Group of the National Administration of Traditional Chinese Medicine of China, Dr. Yanhong Yu pointed out that among the confirmed COVID-19 cases in China, a total of 74,187 people have used Chinese medicine, which accounts to 91.5% of patients (National Administration of Traditional Chinese Medicine, 2020). Academician of Chinese Academy of Engineering Dr. Boli Zhang analyzed 52 patients with COVID-19 retrospectively and found the clinical effective rate of 91.2% in patients treated with integrated traditional Chinese and western medicine as compared to effective rate of 61.1% in patients treated with western medicine alone (Xia et al., 2020).

**Figure 1:**
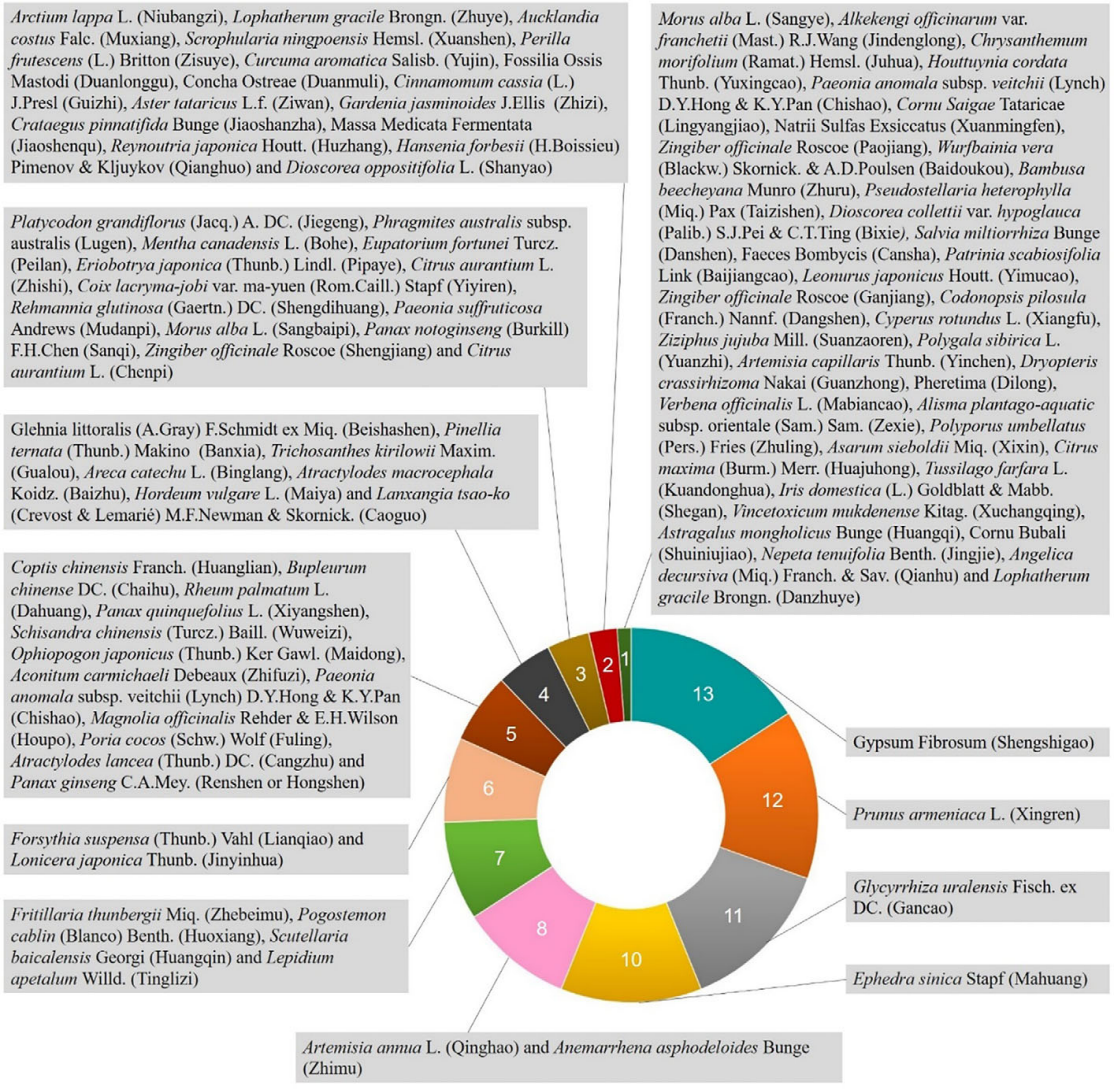
Usage frequency of single herbal medicine in traditional Chinese medicine (TCM) part of the China National Guidelines of Diagnosis and Treatment for SARS-CoV, MERS-CoV, SARS-CoV-2, and influenza virus (The statistical data were based on the analysis of each traditional Chinese herbal medicine involved in different syndromes of viral pneumonia in China’s National Guidelines for TCM treatment of SARS-CoV, MERS-CoV, SARS-CoV-2, and influenza virus).

**Figure 2:**
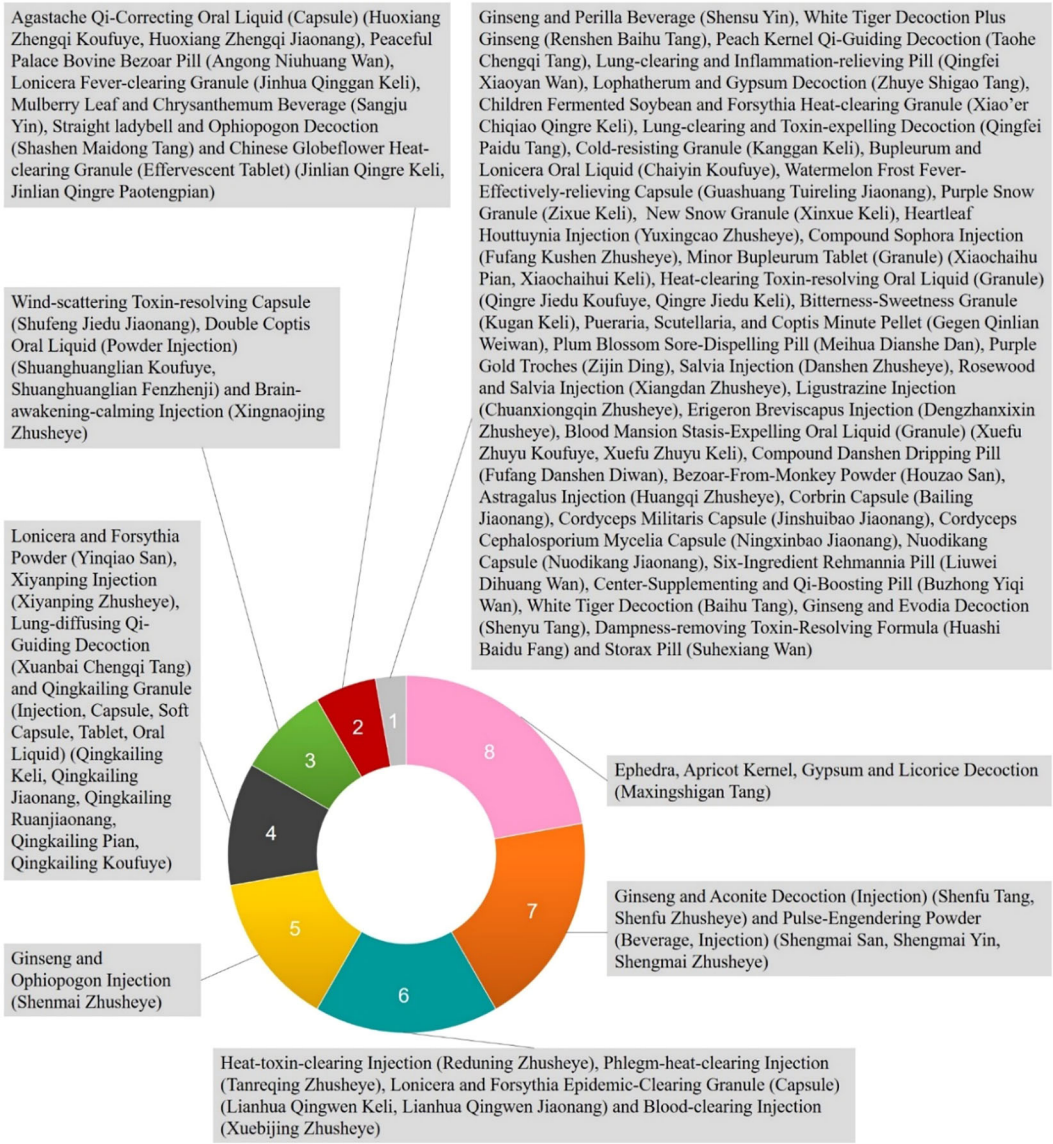
Usage frequency of Chinese medical formulas and proprietary traditional Chinese medicine products in traditional Chinese medicine (TCM) part of the China National Guidelines of Diagnosis and Treatment for SARS-CoV, MERS-CoV, SARS-CoV-2, and influenza virus (The statistical data were based on the analysis of Chinese medical formulas and proprietary traditional Chinese medicine products involved in different syndromes of viral pneumonia in China’s National Guidelines for TCM treatment of SARS-CoV, MERS-CoV, SARS-CoV-2, and influenza virus).

Although there is no name of “viral pneumonia” in TCM, it is mainly attributed to “exogenous diseases” or “exterior syndrome”. Traditional Chinese medical physicians usually classified them as “cough” or “lung distention” according to its clinical manifestations. Moreover, viral pneumonia with strong infectivity and high fatality rate is usually classified as “epidemic disease” in TCM. There has been a long history in China that TCM has been used to treat “epidemic disease” and there are a lot of clinical experiences and excellent efficacy. Therefore, different health organizations in China focus on TCM prevention and treatment of viral pneumonia and have formulated a series of diagnosis and treatment guidelines (China Association of Chinese Medicine, 2003; National Health and Family Planning Commission of People’s Republic of China, 2015; National Health and Family Planning Commission of People’s Republic of China, 2017; National Health Commission of the People’s Republic of China and National Administration of Traditional Chinese Medicine, 2019; National Health Commission of the People’s Republic of China, 2020). Among them, dozens of Chinese herbal medicines and formulae have been proposed (See **Figures 1** and **2**). Single traditional Chinese herbal medicine commonly-used in these diagnosis and treatment guidelines includes *Gypsum Fibrosum* (Shengshigao), *Glycyrrhiza uralensis* Fisch. ex DC. (Gancao), *Prunus armeniaca* L. (Xingren), *Ephedra sinica* Stapf (Mahuang), *Scutellaria baicalensis* Georgi (Huangqin), *Artemisia annua* L. (Qinghao), *Lonicera japonica* Thunb. (Jinyinhua), *Forsythia suspensa* (Thunb.) Vahl (Lianqiao), *Lepidium apetalum* Willd. (Tinglizi), *Anemarrhena asphodeloides* Bunge (Zhimu), *Fritillaria thunbergii* Miq. (Zhebeimu), *Pogostemon cablin* (Blanco) Benth. (Huoxiang) and *Ophiopogon japonicus* (Thunb.) Ker Gawl. (Maidong), etc. The recommended basic medical formulae for the treatment of viral pneumonia include the Ephedra, Apricot Kernel, Gypsum and Licorice Decoction (Maxingshigan Tang), which is used in the highest frequency (China Association of Chinese Medicine, 2003; Wang et al., 2011; National Health Commission of the People’s Republic of China, 2020; Xi et al., 2020). For the main clinical manifestations of viral pneumonia such as fever, cough and panting, *Gypsum Fibrosum* (Shengshigao) can clear and discharge lung-heat, and vent pathogen with acrid-cool (medicinals). *Prunus armeniaca* L. (Xingren) and *Ephedra sinica* Stapf (Mahuang) can diffuse the lung, relieve cough and calm panting. *Glycyrrhiza uralensis* Fisch. ex DC. (Gancao) and *Ephedra sinica* Stapf (Mahuang) have antiviral and immune regulating effect (Mantani et al., 2001; Cinatl et al., 2003). *Lonicera japonica* Thunb. (Jinyinhua), *Forsythia suspensa* (Thunb.) Vahl (Lianqiao), *Artemisia annua* L. (Qinghao), *Pogostemon cablin* (Blanco) Benth. (Huoxiang), and *Scutellaria baicalensis* Georgi (Huangqin) are also commonly used in the recommended prescription and have been reported to possess immunoregulatory and antiviral activities (Efferth et al., 2008; Duan et al., 2012; Shen et al., 2012; Liu F. et al., 2016; Xu et al., 2019).

The role of Chinese herbal medicine in antivirus is usually considered interfering the procession of virus pathogenesis to achieve anti-virus effects, such as suppressing the virus proliferation, preventing the adhesion of virus into susceptible host cells, promoting the immune response, suppressing the excessive abnormal inflammatory response and regulating the immune function of the body. These antiviral effects are often referred to as “detoxification” or “resolving toxins” in the theory of traditional Chinese medicine (Xi and Gong, 2017). In recent years, many researches and progresses have been made to understand the action and mechanism of TCM in the treatment of viral pneumonia for clinical purpose. Through the collection and analysis of nearly 20 years of literature, these actions and mechanisms were discussed from the perspective of direct and indirect antiviral effects as well as immunomodulatory effects in this report.

## Methods

All data were retrieved from the PubMed, Web of Knowledge, China National Knowledge Infrastructure (CNKI), Wanfang Database, VIP Database, China Biology Medicine disc (CBMdisc) and official websites from January 1, 2000 to August 8, 2020 (including), and collected from the TCM diagnosis and treatment literatures and data related to viral pneumonia issued by the National Health Commission of China, the Health Commissions of provinces, autonomous regions and municipalities directly under the central government, the State Administration of Traditional Chinese Medicine of China and the Administration of Traditional Chinese Medicine of all provinces, as well as the opinions expressed from TCM masters, academicians and famous TCM clinical experts through open channels. These date were extracted into two tables by two independent researchers according to the inclusion and exclusion criteria after reaching a consensus. When there are differences in the process of screening and data extraction, it was submitted to the third party for joint decision. The inclusion criteria included the following: *a*. Clear literatures on the experimental and clinical research on the treatment of viral pneumonia with herbs, herbal extracts, or Chinese medical formulae. *b*. Literatures written in English and Chinese. The exclusion criteria were as follows: *a.* Literatures without control medicinals in experimental research; *b.* Literature review, individual case report, expert experience introduction and other types of literature; *c.* For the content of the repeatedly published literature or the repeatedly quoted literature, only one article is included.

## Results

### Experimental Research

Animal or cell studies have found that some traditional Chinese herbal medicines and medical formulas have a variety of pharmacological effects in the treatment of viral pneumonia. In addition to the direct or indirect antiviral effect (See **Tables 1** and **2**), the best advantage of TCM is the regulation of immune function and low adverse effects (Ma et al., 2013).

**Table 1:** Effects and mechanisms of single Chinese medicine and its components on antiviral pneumonia.

Name of Herb	Components contained/product	Model/Strains	Dosage/Duration	IC_50_, EC_50_, TCID_50,_ TC_50_, TC_0_, LD_50,_ CD_50_ *	Control	Actions and mechanisms	References
*Rhus chinensis* Mill. (Wubeizi)	Ethyl acetate extract: acyl pentagalic acid glucose, ellagic acid and gallic acid	*In vitro*: influenza virus neuraminidase (NA)	10, 25, 50, 75, 100 mg/L	62.81 mg/L (IC_50_)	Oseltamivir	Inhibits neuraminidase activity, restrain influenza virus A/PR/8/34 (H1N1), inhibits the activation of TLR4 and the downstream MyD88 dependent transduction pathway, reduces the transcription of inflammatory factors, and alleviates lung inflammation and slow down the process of acute lung injury.	Yang et al., 2017; Zhang et al., 2018
	Ethanol extract: acyl pentagalic acid glucose, ellagic acid and gallic acid			58.31 mg/L (IC_50_)			
	Gallic acid	*In vitro*: A/California/07/2009 (H1N1) in MDCK	2.5 μg/ml	2.6 ± 0.07 μg/ml (EC_50_)	Amantadine		
*Osmunda japonica* Thunb. (Ziqiguanzhong)	Isoginkgetin	*In vitro*: A/Califomia/04/2009 (H1N1)	50 mg/L	42.54 ± 2.85 μmol/L (IC_50_)	Oseltamivir	Inhibits neuraminidase activity, and plays an anti-inflammatory role through IκB and c-JUN pathway.	Lang et al., 2019; Li et al., 2019
*Nepeta tenuifolia* Benth. (Jingjie)	Volatile oil of Nepeta tenuifolia	In vitro: A/PR/8/34 (H1N1) in MDCK	3.1×10^-3^-0.10 mg/ml	3.1×10^-3^ mg/ml (IC_50_); 0.20 mg/ml (TC_50_); 0.10 mg/ml (TC_0_)	Ribavirin	Inhibits and kills influenza virus, increases the content of IFN-α, TLR7, IFN-β and IL-2 and inhibits the secretion of IL-6 and TNF-α in the serum of mice, and restrains the protein expression of Myd88 and TRAF6.	Xie X. H. et al., 2007; He et al., 2012; Gou et al., 2013; He et al., 2013
	Pulegone		1.3×10^-2^-0.10 mg/ml	7.2×10^-3^ mg/ml (IC_50_); 0.36 mg/ml (TC_50_); 0.10 mg/ml (TC_0_)			
	Menthone		7.8×10^-3^-0.10 mg/ml	1.9×10^-3^ mg/ml (IC_50_); 0.43 mg/ml (TC_50_); 0.25 mg/ml (TC_0_)			
*Arctium lappa* L. (Niubangzi)	Arctigenin	*In vitro*: A/FM1/1/47 (H1N1) in MDCK	500, 125, 31.25, 7.81 mg/ml	31.25 mg/ml (IC_50_)	Ribavirin	Inhibits the release of progeny viruses from the host cells and induces interferon *in vivo*; and improves the protein expression of IFN to regulate human immune function.	Tsou, 2007; Fu et al., 2008; Hayashi et al., 2010
*Ilex asprella* (Hook. et Arn.) Champ. ex Benth. (Gangmei)	Water extraction of Ilex asprella root	*In vitro*: A/FM1/1/47 (H1N1) in MDCK	50, 12.5, 3.12, 0.78 mg/ml	23.04 mg/ml (TC_50_)	Ribavirin	Inhibits influenza virus type A FM1 strain, RSV and parainfluenza virus type 3, increases the percentage of CD3+ in peripheral blood T lymphocyte subsets, and regulates the ratio of CD4/CD8 to enhance the immune function of mice.	Chen et al., 2016
	Water extraction of Ilex asprella stem			10.82 mg/ml (TC_50_)			
*Reynoutria japonica* Houtt. (Huzhang)	Resveratrol	*In vitro*: A/PR/8/34 (H1N1) in MDCK	31.25–1,000 mg/L	129.8 μmol/L (IC_50_); 5.9 μmol/L (EC_50_)	Zanamivir	Inhibits neuraminidase activity, reduces lung index, alleviates pulmonary edema, reduces inflammatory factors TNF-α, IL-1β, increases PI3K protein expression, and reduces the protein expression of p-Akt, caspase-3 and NF-κB.	Chen et al., 2012; Li M. et al., 2020
		In vitro: A/Guangdong/243/72 (H3N2) in MDCK		144.7 μmol/L (IC_50_)			
	Catechin-3-O-gallate	*In vitro*: A/PR/8/34 (H1N1) in MDCK		21.3 μmol/L (IC_50_); 0.9 μmol/L (EC_50_)			
		*In vitro*: A/Guangdong/243/72 (H3N2) in MDCK		21.5 μmol/L (IC_50_)			
*Isatis tinctoria* L. (Banlangen)	Isatis root polysaccharide (IRP)	*In vitro*: A/Califomia/04/2009 (H1N1), A/Anhui/1/2005 (H5N1) in Fluorescein-n-acetylneuraminic acid solution	0.5-35 mg/ml	H1N1: 24.13 mg/ml (IC_50_); H5N1: 22.91 mg/ml (IC_50_)	Oseltamivir, apigenin	Inhibits neuraminidase activity in influenza virus H1N1 and H5N1, and decrease the protein expression of IFN-β by downregulating the expression of TLR3, TBK1 and p-IRF3 in RAW264.7 cells infected by respiratory syncytial virus (RSV), attenuates inflammation, and decreases H1N1 viral replications in lungs, reduces the protein expression of mitofusin-2 (MFN2) to reduce the susceptibility to influenza virus *via* mitochondrial antiviral signaling.	Li et al., 2009; Li J, 2013; Hou et al., 2017; Luo et al., 2019
	Acid Isatis root polysaccharide (IRPA)			H1N1: 5.88 mg/ml (IC_50_); H5N1: 4.12 mg/ml (IC_50_)			
	Neutral Isatis root polysaccharide (IRPN)			H1N1: 52.34 mg/ml (IC_50_); H5N1: 49.39 mg/ml (IC_50_)			
	Isatis root polysaccharide (IRP)	*In vitro*: A/Califomia/04/2009 (H1N1), A/Anhui/1/2005 (H5N1) in Fluorescein-4,7-dimethoxy-n-acetylneuraminic acid solution		H1N1: 25.66 mg/ml (IC_50_); H5N1: 23.10 mg/ml (IC_50_)			
	Acid Isatis root polysaccharide (IRPA)			H1N1: 6.09 mg/ml (IC_50_); H5N1: 5.08 mg/ml (IC_50_)			
	Neutral Isatis root polysaccharide (IRPN)			H1N1: 55.19 mg/ml (IC_50_); H5N1: 52.16 mg/ml (IC_50_)			
*Gardenia jasminoides* J. Ellis (Zhizi)	Geniposide, Gardenia extract ZG	*In vitro*: A3/Guifang/81/23 (H3N2) in A549	6.25–1,600 μg/ml	50-60 μg/ml (IC_50_)	Ribavirin	Decreases the protein expression of IL-6, TNFα, TLR3 and NF-κB and mRNA expression of TLR7/MyD88 and TRIF in lung tissue of H3N2-infected mice, and improve cell membrane fluidity.	Guo et al., 2007; Zhang and Yu, 2010; Wang Y. F. et al., 2020
*Hypericum perforatum* L. (Guanyelianqiao)	Hypericin, hyperoside	In vitro: 2337/A/Gansuchengguan/1771/2006 (H1N1) in MDCK	100, 50, 25, 12.5 μg/ml	10^-4.90^/0.1 ml (TCID_50_); 200 μg/ml (TC_0_)	Oseltamivir	Increases the function of T and B lymphocyte conversion, phagocytic function of macrophages and NK killing activity of influenza virus-infected mice, decreases the content of IL-6 and TNF-α and increases the protein expression of IFN-γ and IL-10 in lung tissue and serum of mice.	Wang et al., 2009; Xu et al., 2016
*Andrographis paniculata* (Burm.f.) Nees (Chuanxinlian)	Water extraction of Andrographis paniculata	*In vitro*: A/FM1/1/47 (H1N1) in MDCK	250, 62.5, 15.63, 3.9, 0.98, 0.24 mg/ml	10^-3.50^/0.1 ml (TCID_50_); 250 mg/ml (TC_0_)	Ribavirin	Increases the percentage of CD3+ in peripheral blood T lymphocyte subsets, regulates the ratio of CD4/CD8, enhances the immune function of mice, and shows antiviral activity against H5N1 virus.	Sornpet et al., 2017; Wang et al., 2019
	Ethanol extraction of Andrographis paniculata	*In vitro*: influenza virus A/Chicken/Thailand/CUK2/04 (H5N1) in MDCK	8.2 g/ml	8.2 μg/ml (CD_50_)	–		
	Water extraction of Andrographis paniculata			380.3 μg/ml (CD_50_)			
*Lonicera japonica* Thunb. (Jinyinhua)	Chlorogenic acid, caffeic acid	In vitro: influenza A (H3N2) in MDCK	5 mg/ml	236.28 ± 15.37 μg/ml (IC_50_)	Ribavirin	Inhibits the influenza virus proliferation and neuraminidase activity, increases the content of IFN-γ in serum, and decrease the lung index.	Shen et al., 2012; Zhu et al., 2018; Zhao et al., 2020
		In vitro: Influenza A (H1N1) in MDCK		290.50 ± 34.82 μg/ml (IC_50_)			
*Scutellaria baicalensis* Georgi (Huangqin)	Baicalin	In vitro: A/FM1/1/47 (H1N1) in MDCK/A549	20, 30, 40, 60, 80 μg/ml (in MDCK); 5, 10, 20, 30, 40 μg/ml (in A549)	43.3/40.3 μg/ml (EC_50_)	Ribavirin	Inhibits neuraminidase activity, reduces virus replication, and decreases the protein expression of TLR3 and NF-κB and mRNA expression of TRIF, the protein and gene expression of proinflammatory cytokines TNF-α, IL-1 and IL-6 in lung tissue, and increases the protein and gene expression of anti-inflammatory cytokine IL-10 and antiviral factor IFN-γ in lung tissue after infection.	Ding et al., 2014; Wang et al., 2014; Zhang and Yu, 2010; Xu et al., 2019
		In vitro: A/Beijing/32/92 (H3N2) in MDCK or A549		104.9/100.1 μg/ml (EC_50_)			
		*In vivo*: A/FM1/1/47 (H1N1) in mice	50, 100, 200 mg/kg/day	52.3 μmol/L (IC_50_)			
		*In vivo*: A/Beijing/32/92 (H3N2) in mice		85.8 μmol/L (IC_50_)			
*Coptis chinensis* Franch. (Huanglian) and *Magnolia officinalis* Rehder & E.H.Wilson (Houpo)	Berberine	In vitro: influenza virus neuraminidase (NA)	32, 16, 8.0, 4.0, 2.0, 1.0, 0.5 mg/ml	21.10 mg/ml (IC_50_)	Oseltamivir	Inhibits neuraminidase activity, restrain influenza A (H1N1) virus, reduce the lung index of infected mice and ameliorates the lung pathological changes, suppresses the viral infection‐induced up‐regulation of TLR7 signaling pathway, such as TLR7, MyD88, and NF-κB (p65), at both the mRNA and protein levels, and inhibits the viral infection‐induced increase in Th1/Th2 and Th17/Treg ratios as well as the production of inflammatory cytokines.	Wu et al., 2014; Chen et al., 2017; Enkhtaivan et al., 2018; Yan et al., 2018
	Berberine: magnolol (1:5)			19.09 mg/ml (IC_50_)			
	Berberine: magnolol (2:5)			15.39 mg/ml (IC_50_)			
	Berberine: magnolol (2:3)			3.80 mg/ml (IC_50_)			
	Berberine: magnolol (1:1)			3.53 mg/ml (IC_50_)			
	Berberine: magnolol (3:2)			13.66 mg/ml (IC_50_)			
	Berberine: magnolol (5:2)			8.90 mg/ml (IC_50_)			
	Berberine: magnolol (5:1)			19.04 mg/ml (IC_50_)			
	Magnolol			19.37 mg/ml (IC_50_)			
	Berberine: magnolol (1:1)	*In vivo*: A/PR/8/34 (H1N1) in mice	8 g/kg	10^-2.5^/100 μl (LD_50_)	Ribavirin		
*Ephedra sinica* Stapf (Mahuang)	(+) - Catechin	In vitro: A/California/07/2009 (H1N1) in MDCK	25 μg/ml	18.4 ± 0.7 μg/ml (EC_50_)	Amantadine	Suppresses the proliferation of influenza virus H1N1 and neuraminidase activity, and inhibits the adsorption and penetration of respiratory syncytial virus.	Mantani et al., 2001; You et al., 2018; Zhu, 2008
	Water extract of Ephedra sinica	*In vitro*: respiratory syncytial virus (RSV) in Hela	5.00, 4.00, 3.20, 2.56 mg/ml	3.74 mg/ml (EC_50_)	Ribavirin		
*Forsythia suspensa* (Thunb.) Vahl (Lianqiao)	Ethanol extract: Forsyshiyanins A-B, Forsythiaside (Phillyrin)	*In vitro*: A/PR/8/34 (H1N1) in MDCK	10 μM	18.4-26.2 μM (IC_50_)	Ginkgolide B	Forsyshiyanins A-B inhibit NP gene expression of influenza A virus after transfection. Forsythoside A reduces the viral titers of different influenza virus subtypes in cell cultures and increases the survival rate of the mice in an *in vivo* influenza virus infection model, and reduces the influenza M1 protein, which in turn intervenes the budding process of the newly formed virions.	Duan et al., 2012; Law et al., 2017; Zhao et al., 2020
		*In vitro*: respiratory syncytial virus (RSV) Long in Hep-2		10.5-14.4 μM (EC_50_)			
*Morus alba* L. (Sangbaipi)	Cortex mori polysaccharide, total flavonoids of Cortex mori	*In vitro*: respiratory syncytial virus (RSV) Long in Hep-2	–	10^-2.25^/100 μl (TCID_50_)	Ribavirin	Inhibits respiratory syncytial virus, reduces the infiltration of inflammatory cells in alveolar wall to ameliorate the inflammatory status of lung tissue, promotes cell immune adjustment in mice infected by respiratory syncytial virus, decreases the protein expression of PI3K, Akt1/2 and NF-κBp65 in lung tissue of mice as well as the IL-4 and INF-γ in serum.	Dong et al., 2016a; Dong et al., 2016b; Liu, 2016
	Cortex mori polysaccharide	*In vivo*: respiratory syncytial virus (RSV) Long in mice	91 mg/kg/day	10^-1.92^/100 μl (LD_50_)			
	Total flavonoids of Cortex mori		114 mg/kg/day				
*Houttuynia cordata* Thunb. (Yuxingcao)	Quercetin, isoquercetin	*In vitro*: Influenza A (H3N2) in MDCK	5 mg/ml	428.97 ± 38.54 μg/ml (IC_50_)	Ribavirin	*Houttuynia cordata* Thunb. extract, quercetin and cinanserin inhibit the activity of murine coronavirus and the protein expression of NF-κB, and restrains the replication of influenza A virus *in vitro*. Quercitrin inhibites both viral replication and TLR signaling in cells. Flavonoids from Houttuynia cordata attenuate H1N1-induced acute lung injury in mice *via* inhibition of influenza virus and Toll-like receptor signaling.	Chiow et al., 2016; Zhu et al., 2018; Ling et al., 2020
		*In vitro*: Influenza A (H1N1) in MDCK		522.28 ± 36.48 μg/ml (IC_50_)			
	Ethyl acetate (EA) fraction of *Houttuynia cordata* Thunb.	*In vitro*: Murine coronavirus	0.24-3.91 mg/ml	0.98 μg/ml (IC_50_)	Rutin		
	Quercetin		15.63-500.00 mg/ml	125.00 μg/ml (IC_50_)			
	Cinanserin (1 dpi)Cinanserin (2 dpi)		3.91-125.00 mg/ml	31.25 μg/ml (IC_50_)62.50 μg/ml (IC_50_)			
*Cinnamomum cassia* (L.) J.Presl (Guizhi)	Cinnamic aldehyde	*In vitro*: A/PR/8/34 (H1N1) in MDCK	0.132, 0.264 mg/kg	5.31×10^-5^ mg/ml (IC_50_)	Ribavirin	Inhibits the proliferation of influenza A virus (H1N1) in MDCK cells, restrains the infection by interfering with endocytosis, kills influenza virus, and increases the content of IFN-α and IFN-β in the serum of H1N1-infected mice.	Liu et al., 2012; Gou et al., 2013; Liu et al., 2013; Zhuang et al., 2009
	Volatile oil of Cassia twig		0.174, 0.348 mg/kg	5.80×10^-5^ mg/ml (IC_50_)			
	Cinnamomi Cortex extract	*In vitro*: wild-type SARS-CoV in Vero E6 cells	0.1, 0.2, 0.3 mg/ml	43.1± 2.8 μg/ml (IC_50_)	–		
	Ethanol extract of Cinnamomi Cortex			10.7± 0.4 μg/ml (IC_50_)			
	Butanol fraction of Cinnamomi Cortex			7.8± 0.3 μg/ml (IC_50_)			
	Aqueous fraction of Cinnamomi Cortex			39.7± 2.1 μg/ml (IC_50_)			
*Syzygium aromaticum* (L.) Merr. & L.M.Perry (Dingxiang)	Methanol extraction of Clove	*In vitro*: A/PR/8/34 (H1N1) in MDCK	30, 10, 10/3, 10/9, 10/27, 10/81 μg/ml	9.1 μg/ml (IC_50_)	Zanamivir, ribavirin	Inhibits neuraminidase activity, restrains the infection by interfering with endocytosis, increases the levels of IFN-γ and IL-10 in serum, decreases the levels of TNF-α and IL-6, alleviates the pathological changes of lung tissue and decreases the lung index of mice infected by A/swine/Tianjing/14/2009(H1N1).	Zhuang et al., 2009; Li et al., 2013; Liu et al., 2018
	Eugeniin			7.85 μg/ml (IC_50_)			
	CFE (Caryophylli Flos extract)	*In vitro*: wild-type SARS-CoV in Vero E6 cells	0.1, 0.2, 0.3 mg/ml	50.1± 3.5 μg/ml (IC_50_)	–		
*Cibotium barometz* (L.) J.Sm. (Gouji)	Ethanol extraction of Cibotium barometz	In vitro: SARS-CoV in Vero E6 cells	200, 100, 50, 25 μg/ml	8.42 μg/ml (EC_50_)	Valinomycin	Inhibits viral replication and the enzymatic activity of SARS-CoV 3CL protease, and inhibit respiratory syncytial virus induced cytopathic effect *in vitro*.	Li et al., 2008; Wen et al., 2011
	Methanol extraction of Cibotium barometz			>10 μg/ml (EC_50_)			
*Taxillus chinensis* (DC.) Danser (Sangjisheng)	N-hexane extraction of Taxillus chinensis			5.39 μg/ml (EC_50_)			
*Dioscorea oppositifolia* L. (Shanyao)	Methanol extraction of Dioscorea batatas			8.06 μg/ml (EC_50_)			
*Senna tora* (L.) Roxb. (Juemingzi)	N-hexane extraction of Cassia tora			8.43 μg/ml (EC_50_)			
*Gentiana scabra* Bunge (Longdan)	N-hexane extraction of Gentiana scabra			8.70 μg/ml (EC_50_)			
	Active compound RG2-1 from Gentiana scabra	*In vitro*: respiratory syncytial virus (RSV) Long in Hela cells	50, 25, 12.5, 6.25 mg/ml	11.07 mg/ml (TC_50_); 0.42 mg/ml (EC_50_)	Ribavirin		
*Glycyrrhiza uralensis* Fisch. ex DC. (Gancao)	Glycyrrhizic acid	In vitro: SARS-associated coronavirus in Vero cells	1,000, 4,000 mg/ml	After virus adsorption: 600 mg/L (EC_50_); during and after virus adsorption: 300 mg/L (EC_50_); during virus adsorption: 2,400 mg/L (EC_50_)	Mycophenolic acid, pyrazofurin and ribavirin	Inhibits virus replication, adsorption and membrane penetration, up-regulates the mRNA expression of IFN-γ and its immunomodulatory function against influenza virus infection, down-regulates the mRNA expression of TNF-α, and decreases the inflammatory reaction induced by TNF-α to reduce the host immune damage.	Cinatl et al., 2003; Xie Z. P. et al., 2007; Liu et al., 2006; Fu X. L. et al., 2020
	Active compound (GC3-1-4)	*In vitro*: Parainfluenza virus (type III) in Hela	25, 20, 16,12.8, 10.2 μg/ml	12.82 μg/ml (EC_50_); 144.17 μg/ml (TC_50_)	Ribavirin		
		*In vitro*: respiratory syncytial virus (RSV) in Hela	100, 75, 56, 42, 32, 24 μg/ml	41.32 μg/ml (EC_50_); 0.45 mg/ml (TC_50_)			
*Rheum officinale* Baill. (Dahuang)	Emodin (1,3,8-trihydroxy-6-methylanthraquinone), aqueous extract of Radixet Rhizoma Rhei	I*n vitro*: SARS-CoV in Vero E6 cells	0.1, 0.5, 1, 5, 10, 50, 100 μg/ml	200 μM (IC_50_)	Promazine	Blocks the S protein and ACE2 interaction in a dose-dependent manner and also inhibits the infectivity of S protein-pseudotyped retrovirus to Vero E6 cells.	Ho et al., 2007

*IC_50_, 50% inhibiting concentration; EC_50_, 50% maximal effective concentration; TCID_50_, 50% tissue culture infectious dose; TC_50_, median toxic concentration. TC_O,_ Maximum nontoxic concentration; LD_50_, median lethal dose; CD_50_, 50% cytotoxicity dose.

**Table 2:** Effects and mechanisms of Chinese medical formulas on antiviral pneumonia.

Name of Formulas	Ingredients	Efficacy	Usage Mode	Model/Strains (Registration number)	Dosage/Duration	IC_50_, EC_50_, TCID_50,_ TC_50_, TC_0_, LD_0_, LD_50_*	Control	Actions and mechanisms	References
****Ephedra Decoction (Mahuang Tang)	*Ephedra sinica* Stapf (Mahuang) 9g, Cinnamomum cassia (L.) J. Presl (Guizhi) 6g, Prunus armeniaca L. (Xingren) 6g and Glycyrrhiza uralensis Fisch. ex DC. (Gancao) 3g.	Induce sweating to release the exterior, diffuse the lung and relieve panting.	Decoction	*In vitro*: influenza virus A/PR/8/34 (H1N1) in MDCK	5.0, 2.5, 1.25, 0.63, 0.31 g/L	1.59 g/L (EC_50_); 61.66 g/L (TC_50_); 5.66 g/L (TC_0_)	Oseltamivir	Blocks the invasion of influenza virus into host cells, inhibits the biosynthesis of influenza virus in cells, and down-regulates the expression levels of TLR4, TLR7, MyD88 and TRAF6 mRNA in cells.	Wei et al., 2018
Three Substances Scutellaria Decoction (Sanwu Huangqin Tang)	*Sophora flavescens* Aiton (Kushen) 6g, *Scutellaria baicalensis* Georgi (Huangqin) 6g and *Rehmannia glutinosa* (Gaertn.) DC. (Gandihuang) 12g.	Clear heat and resolve toxins, nourish the blood and enrich *yin*.	Extract by water extraction and alcohol sedimentation	*In vitro*: influenza virus A/PR/8/34 (H1N1) in MDCK	0.06, 0.12, 0.24, 0.49, 0.98, 1.95 mg/ml	10^−7^/100 μl (TCID_50_); 12.76 mg/ml (TC_50_); 1.95 mg/ml (TC_0_)	Oseltamivir	Inhibits inﬂuenza A/PR/8/34 (H1N1) virus at different stages of viral replication *in vitro* and *in vivo*.	Ma et al., 2018
				*In vivo*: influenza virus A/PR/8/34 (H1N1) in mice	5.85, 11.70, 23.40 g/kg/day	10^−4.5^/50 μl (LD_50_)			
Pueraria Decoction (Gegen Tang)	Pueraria montana var. lobata (Willd.) Maesen & S.M.Almeida ex Sanjappa & Predeep (Gegen) 96g, *Ephedra sinica* Stapf (Mahuang) 72g, Cinnamomum cassia (L.) J. Presl (Guizhi) 48g, Paeonia lactiflora Pall. (Baishao) 48g, Zingiber officinale Roscoe (Shengjiang) 72g, Ziziphus jujuba Mill. (Dazao) 176g and Glycyrrhiza uralensis Fisch. ex DC. (Gancao) 48g.	Induce sweating to release the exterior, promote fluid production and unblock the channels.	Water extraction of Pueraria Decoction	In vitro: influenza virus (H1N1) in MDCK	6.25 mg/ml	1×10^4.7^/100 μl (TCID_50_); 1.81 mg/ml (IC_50_); 10.88 mg/ml (TC_50_); 6.25 mg/ml (TC_0_)	Oseltamivir	Antagonizes the activity of H1N1 influenza virus, inhibits virus adsorption, restrain the expression of pro-inflammatory factors IL-1α, IL-6 and TNF-α, and downregulates TLR7 expression.	Geng et al., 2019
			Decoction	*In vivo*: influenza virus (H1N1) in mice	1 g/ml	1×10^3.8^/20 μl (LD_50_)			
Heat-toxin-clearing Injection (Reduning Zhusheye) (Patent medicine)	Artemisia annua L. (Qinghao), Lonicera japonica Thunb. (Jinyinhua) and Gardenia jasminoides J.Ellis (Zhizi).	Clear heat, scatter wind and resolve toxins.	Preparation solution of injection	*In vitro*: A/PR/8/34 (H1N1) in MDCK	400 μg/ml	46.49 ± 2.25 μg/ml (IC_50_)	Zanamivir	Inhibits neuraminidase activity of H1N1, H3N2 and B influenza, relieves symptoms such as panting, cough and short of breath, and decreases body temperature.	Sun et al., 2014; Li, 2013
				In vitro: A/Sydney/5/97 (H3N2) in MDCK		49.77 ± 1.77 μg/ml (IC_50_)			
				*In vitro*: B/Jiangsu/10/2003(B) in MDCK		45.33 ± 5.32 μg/ml (IC_50_)			
			Injection	Controlled study *in vivo*: children with viral pneumonia	0.5-0.6 ml/(kg.day)	–	Ribavirin		
Wind-scatering and Lung-diffusing Formula Granule (Shufeng Xuanfei Fang Keli) (Hospital preparation)	Lonicera japonica Thunb. (Jinyinhua), Forsythia suspensa (Thunb.) Vahl (Lianqiao), Persicaria tinctoria (Aiton) Spach (Daqingye), Arctium lappa L. (Niubangzi), Isatis tinctoria L. (Banlangen), Periostracum Cicadae (Chantui), Fritillaria thunbergii Miq. (Zhebeimu), Scutellaria baicalensis Georgi (Huangqin), Nepeta tenuifolia Benth. (Jingjie), Glycine max (L.) Merr. (Dandouchi), Imperata cylindrica (L.) P.Beauv. (Baimaogen) and Glycyrrhiza uralensis Fisch. ex DC. (Gancao).	Clear heat and resolve toxins, and vent the exterior with acrid-cool (medicinals).	Preparation solution of granule	*In vitro*: influenza virus A1/Qianfang/166/85 (H1N1) in A549	10, 5, 2.5, 1.25, 0.63 μg/ml	10^-3.78^/0.1 ml (TCID_50_); 10.20 μg/ml (TC_0_); 38.56 μg/ml (TC_50_); 2.36 μg/ml (IC_50_)	Oseltamivir	Reduces the mortality of mice infected with virus and prolong the average survival time of mice, and downregulates the expression of TLR3, TLR7, MyD88 and IL-6, and increases the expression of IL-4 and IFN-γ.	Liu et al., 2014; Ge et al., 2015; Zhang et al., 2015
			Granular solution	*In vivo*: influenza virus A1/Qianfang/166/85 (H1N1) in mice	3.24, 1.62, 0.81 g/kg	–			
Exterior-releasing and Interior-clearing Formula Granule (Jiebiao Qingli Fang Keli) (Hospital preparation)	Ephedra sinica Stapf (Mahuang), Perilla frutescens (L.) Britton (Zisuye), Nepeta tenuifolia Benth. (Jingjie), Angelica biserrata (R.H.Shan & C.Q.Yuan) C.Q.Yuan & R.H.Shan (Duhuo), Hansenia forbesii (H.Boissieu) Pimenov & Kljuykov (Qianghuo), Gypsum Fibrosum (Shigao), Artemisia annua L. (Qinghao), Scutellaria baicalensis Georgi (Huangqin), Aster tataricus L.f. (Ziwan), Prunus armeniaca L. (Xingren), Platycodon grandiflorus (Jacq.) A. DC. (Jiegeng) and Glycyrrhiza uralensis Fisch. ex DC. (Gancao).	Release the exterior and clear the interior.	Granular solution	*In vitro*: influenza virus A1/Qianfang/166/85 (H1N1) in A549	10, 5, 2.5, 1.25, 0.63 μg/ml	10^-3.78^/0.1 ml (TCID_50_); 9.91 μg/ml (TC_0_); 38.88 μg/ml (TC_50_); 2.46 μg/ml (IC_50_)	Oseltamivir	Downregulates the protein expression of TLR7 and NF-κB, prolongs the average survival time of mice, decreases the mRNA and protein over-expressions of IL-1, TNF-α, IL-6, MCP-1, reduces inflammation, and restores stability and balance of immune function.	Liu et al., 2014; Ge et al., 2015; Zhang et al., 2015
				*In vivo*: influenza virus A1/Qianfang/166/85 (H1N1) in mice	3.82, 1.91, 0.96 g/kg	–			
Lonicera, Forsythia, Bupleurum and Cinnamon Twig Granule (Yinqiaochaigui Keli) (Hospital preparation)	Lonicera japonica Thunb. (Jinyinhua), Forsythia suspensa (Thunb.) Vahl (Lianqiao), Bupleurum chinense DC. (Chaihu), Cinnamomum cassia (L.) J.Presl (Guizhi), Platycodon grandiflorus (Jacq.) A. DC. (Jiegeng), Paeonia lactiflora Pall. (Baishao), Scutellaria baicalensis Georgi (Huangqin), Ephedra sinica Stapf (Mahuang), *Paris polyphylla* var. chinensis (Franch.) H.Hara (Chonglou) and Scrophularia ningpoensis Hemsl. (Xuanshen).	Harmonize ying and wei levels, release the exterior and clear heat toxin.	Granular solution diluted in DMEM	*In vitro*: influenza virus H1N1 (FM1) in MDCK	5, 2.5, 1.25, 0.625, 0.312, 0.156, 0.078, 0.039 mg/ml	2.18 mg/ml (TC_50_); 0.52 mg/ml (IC_50_)	Ribavirin	Decreases the content of TNF-α, IL-6 in BALF, increases the content of SOD in lung homogenate, decreases the content of MDA, and increases the ratio of T lymphocyte subsets, and inhibits TLR7-MyD88-NF-κB signaling pathway.	Xu, 2014; Li et al., 2018
				*In vitro*: influenza virus H1N1 (PR8) in MDCK		3.88 mg/ml (TC_50_); 1.08 mg/ml (IC_50_)			
				*In vitro*: influenza virus A III in MDCK		3.39 mg/ml (TC_50_); >5 mg/ml (IC_50_)			
Sweet Wormwood and Scutellaria Gallbladder-Clearing Decoction (Haoqin Qingdan Tang)	Artemisia annua L. (Qinghao) 6g, Scutellaria baicalensis Georgi (Huangqin) 6g, Citrus aurantium L. (Zhiqiao) 5g, Bambusa tuldoides Munro (Zhuru) 9g, Citrus aurantium L. (Chenpi) 5g, Pinellia ternata (Thunb.) Makino (Banxia) 5g, Poria cocos (Schw.) Wolf (Fuling) 9g, Talcum (Huashi) 6g, Glycyrrhiza uralensis Fisch. ex DC. (Gancao) 1g and Isatis tinctoria L. (Qingdai) 2g.	Clear gallbladder heat and drain dampness, dissolve phlegm and harmonize the stomach.	Preparation solution of decoction	*In vitro*: influenza virus A1/Jingke 96-25, A3/Jingke 92-32, B/Jingfang 93-184 in allantoic cavity of chicken embryo	0.22, 0.12, 0.06 g/ml	2 g/ml (LD_0_)	Shuanghuanglian Oral Liquid	Inhibits influenza virus, and improves lung index and pathological changes and reduces the mRNA expression of NF-κB.	Mo et al., 2005; Lai et al., 2011; Sang et al., 2014
			Decoction	*In vivo*: influenza virus (H1N1) in mice	36.92 mg/(kg.day)	–	Ribavirin		
				Randomized controlled study: patients with influenza viral pneumonia	0.18 g/ml, 300ml/day				
Ascending and Descending Powder (Shengjiang San)	Periostracum Cicadae (Chantui) 10g, Bombyx Batryticatus (Jiangcan) 10g, *Rheum officinale* Baill. (Shengdahuang) 6g and Curcuma longa L. (Jianghuang) 9g.	Clear heat and resolve toxins, dissolve phlegm and dissipate blood stasis.	Decoction	*In vivo*: influenza virus FM1 in mice	4.55 g/kg	10^−3.5^/100 μl (LD_50_)	Ribavirin	Inhibits ICAM-1 and NF-κB overexpression in mouse lung, increases SIgA secretion and the expression of IL-10 and IL-1Rα, and decreases content of IL-1β, IL-6, and TNF-α.	Nan et al., 2016a; Nan et al., 2016b
Wind-scattering Toxins-resolving Capsule (Shufeng Jiedu Jiaonang) (Patent medicine)	Reynoutria japonica Houtt. (Huzhang), Forsythia suspensa (Thunb.) Vahl (Lianqiao), Isatis tinctoria L. (Banlangen), Bupleurum chinense DC. (Chaihu), Patrinia scabiosifolia Link (Baijiangcao), Verbena officinalis L. (Mabiancao), Phragmites australis subsp. australis (Lugen) and Glycyrrhiza uralensis Fisch. ex DC. (Gancao).	Scatter wind and clear heat, resolve toxins and relieve sore throat.	Solution after capsule dissolution	*In vitro*: influenza virus H1N1 (FM1, PR8, Jiangxixiushui, B10, B59), RSV, parainfluenza virus (Xiantai) in A549	5.55, 2.94, 1.52, 0.77 g/L	FM1: 0.56 g/L (IC_50_); PR8: 0.56 g/L (IC_50_); Jiangxixiushui: 0.56 g/L (IC_50_); Brisbane-10: 0.56 g/L (IC_50_); Brisbane-59: 0.56 g/L (IC_50_); RSV: 0.32 g/L (IC_50_); parainfluenza virus (Xiantai): 0.14 g/L (IC_50_)	Ribavirin	Inhibits the protein expression of PGE2, TNF-α, IL-1α, IL-1β, and IL-6, and restrains novel coronavirus by inhibiting MAPK/NF-κB signaling pathway.	Bao et al., 2019; Qu X. K. et al., 2020
Epidemic-pathogen-antagonizing Beverage (Kangli Yin) (Hospital preparation)	Artemisia annua L. (Qinghao) 10g, Scutellaria baicalensis Georgi (Huangqin) 15g, Pinellia ternata (Thunb.) Makino (Banxia) 10g, Periostracum Cicadae (Chantui) 6g, Bombyx Batryticatus (Jiangcan) 10g, Citrus aurantium L. (Chenpi) 5g, Citrus aurantium L. (Zhishi) 10g, Bambusa tuldoides Munro (Zhuru) 10g, Poria cocos (Schw.) Wolf (Fuling) 20g, Curcuma longa L. (Jianghuang) 10g, Pogostemon cablin (Blanco) Benth. (Huoxiang) 10g, *Rheum officinale* Baill. (Dahuang) 5g, Houttuynia cordata Thunb. (Yuxingcao) 15g, Eupatorium fortunei Turcz. (Peilan) 10g and Glycyrrhiza uralensis Fisch. ex DC. (Zhigancao) 5g.	Clear heat and drain dampness, harmonize the stomach and dissolve phlegm.	Decoction solution diluted in DMEM	*In vitro*: influenza virus H1N1 (FM1) in MDCK	22, 11, 5.5, 2.75, 1.375, 0.6875, 0.344, 0.172 mg/ml	9.38 mg/ml (TC_0_); 21.33 mg/ml (TC_50_); >22 mg/ml (IC_50_)	Ribavirin	No significant inhibitory effect on influenza virus H1N1 and RSV Long *in vitro*.	Deng, 2006; Sun, 2006
				In vitro: influenza virus RSV Long in MRC-5	7.62, 3.81, 1.905, 0.953, 0.476, 0.238, 0.119, 0.0595 mg/ml	5.50 mg/ml (TC_0_); 7.62 mg/ml (TC_50_); >7.62 mg/ml (IC_50_)			
			Decoction solution	*In vivo*: influenza virus H1N1 (FM1) in mice	5 g/kg, 2.5 g/kg	–		Reduces lung tissue lesions, increases the levels of CD3+, CD4+, IL-2 and IFN-γ, and inhibits the excessive production of CD8+, IL-6 and TNF-α.	
Two Roots Lung-clearing Beverage (Ergen Qingfei Yin)	Imperata cylindrica (L.) P. Beauv. (Baimaogen) 20g, Phragmites australis subsp. australis (Lugen) 20g, Scutellaria baicalensis Georgi (Huangqin) 10g, Gypsum Fibrosum (Shengshigao) 30g, Prunus armeniaca L. (Xingren) 10g, Ephedra sinica Stapf (Zhimahuang) 6g, Anemarrhena asphodeloides Bunge (Zhimu) 10g, Taraxacum mongolicum Hand.-Mazz. (Pugongying) 10g, Isatis tinctoria L. (Banlangen) 15g, Morus alba L. (Sangbaipi) 10g, Houttuynia cordata Thunb. (Yuxingcao) 10g, Platycodon grandiflorus (Jacq.) A. DC. (Jiegeng) 10g and Aster tataricus L.f. (Ziwan) 10g.	Clear heat and dissolve phlegm, diffuse the lung and direct lung qi downward.	Decoction	Randomized controlled study with random number table: patients with influenza A (H1N1) viral pneumonia	0.923 g/ml, 200 ml/day	–	Oseltamivir	Relieves the symptoms of cough, expectoration, dry mouth, vexation, fever, panting and pulmonary rales, increases the level of IL-10 and decreases the level of IL-6, IL-8, TNF-α and C-reactive protein in serum.	Tian et al., 2019
Sweet Dew Toxin-Removing Elixir (Ganlu Xiaodu Dan)	Talcum (Huashi) 45g, *Scutellaria baicalensis* Georgi (Huangqin) 30g, Artemisia capillaris Thunb. (Yinchen) 30g, Acorus calamus var. angustatus Besser (Shichangpu) 18g, Fritillaria cirrhosa D.Don (Chuanbeimu) 15g, *Akebia trifoliata* (Thunb.) Koidz. (Mutong)15g, Pogostemon cablin (Blanco) Benth. (Huoxiang) 12g, Forsythia suspensa (Thunb.) Vahl (Lianqiao) 12g, *Wurfbainia vera* (Blackw.) Skornick. & A.D.Poulsen (Baidoukou) 12g, *Mentha canadensis* L. (Bohe) 12g and Iris domestica (L.) Goldblatt & Mabb. (Shegan) 12g.	Drain dampness and remove turbidity, clear heat and resolve toxins.	Decoction	*In vivo*: influenza virus A/PR/8/34 (H1N1) in mice	56 g/kg/day	–	Ribavirin	Decrease the level of serum IL-4 and expression of H1N1 mRNA and Aquaporin 1 (AQP1) protein in mice model with viral pneumonia.	Bi et al., 2019; Chen L. et al., 2020
				Retrospective study: patients with SARS-CoV-2 infection	0.71 g/ml	–	Arbidol, Moxifloxacin		
Folium Ginkgo and Ephedra Lung-clearing Capsule (Yinhuang Qingfei Jiaonang) (Patent medicine)	Lepidium apetalum Willd. (Beitinglizi), Ephedra sinica Stapf (Mahuang), Prunus armeniaca L. (Kuxingren), Fritillaria thunbergii Miq. (Zhebeimu), Eriobotrya japonica (Thunb.) Lindl. (Pipaye), Persicaria tinctoria (Aiton) Spach (Daqingye), Acorus calamus var. angustatus Besser (Shichangpu), Dioscorea nipponica Makino (Chuanshanlong), Aconitum brachypodum Diels (Yizhihao), Ginkgo biloba L. (Yinxingye), Schisandra chinensis (Turcz.) Baill. (Wuweizi), Citrus aurantium L. (Zhishi), Gypsum Fibrosum (Shengshigao) and Glycyrrhiza uralensis Fisch. ex DC. (Gancao).	Clear lung heat and dissolve phlegm, relieve cough and calm panting.	Solution after capsule dissolution	*In vivo*: A/PR/8/34 (H1N1) in rat	100, 200, 400 mg/kg	–	Bairui Capsule	Inhibits neuraminidase activity and influenza virus proliferation *in vivo*, improves pneumonia symptoms and histopathological changes, and increases the level of FGF2 and protein expression of FGFR1 in the lung.	Li et al., 2015; Peng et al., 2016; Qiu et al., 2018
			Medicated serum	*In vitro*: A/PR/8/34 (H1N1) in MDCK	50%, 25%, 12.5%, 6.25%, 3.125% (serum concentration)	25% of the containing serum solution (TC_0_)	Ribavirin		
				*In vitro*: respiratory syncytial virus (RSV) in Hep-2	12.5%, 6.25%, 3.13%, 1.56%, 0.78% (serum concentration)	12.5% of the containing serum solution (TC_0_); 10^-8^/0.1 ml (TCID_50_)			
Ephedra, Apricot Kernel, Gypsum and Licorice Decoction (Maxingshigan Tang)	Ephedra sinica Stapf (Mahuang) 6g, Prunus armeniaca L. (Xingren) 6g, Gypsum Fibrosum (Shengshigao) 24g and Glycyrrhiza uralensis Fisch. ex DC. (Gancao) 6g.	Release the exterior with acrid-cool (medicinals), clear lung heat and relieve panting.	Decoction	*In vivo*: respiratory syncytial virus (RSV) Long in rat	1.87, 2.8, 4.2, 6.3 g/kg	3.992 g/kg (ED_50_)	Ribavirin	Inhibits neuraminidase activity and influenza virus proliferation.	Hsieh et al., 2012; Cui et al., 2019
Lung-clearing and Collaterals-unblocking Ointment (Qingfei Tongluo Gao) (Hospital preparation)	*Rheum officinale* Baill. (Dahuang) and Scutellaria baicalensis Georgi (Huangqin) and *Allium sativum* L. (Dasuan).	Clear heat and resolve toxins.	Paste	*In vivo*: respiratory syncytial virus (RSV) Long in rat	0.6 g/paste	10^-3^/0.1 ml (TCID_50_)	–	Reduces the scope of lung lesions and alveolar exudates, and inhibits PI3K/Akt/NF-κB signaling pathway in intervention of RSV pneumonia.	Liu X. X. et al., 2016; Zhang et al., 2016
Lung-clearing Oral Liquid (Qingfei Koufuye)	Ephedra sinica Stapf (Mizhimahuang) 4g, Prunus armeniaca L. (Kuxingren) 10g, Angelica decursiva (Miq.) Franch. & Sav. (Qianhu) 10g, Gypsum Fibrosum (Shengshigao) 24g, Morus alba L. (Mizhisangbaipi), Lepidium apetalum Willd. (Tinglizi) 6g, Bistorta officinalis Delarbre (Quanshen) 12g, Bombyx Batryticatus (Baijiangcan) 6g, Reynoutria japonica Houtt. (Huzhang) 12g and Salvia miltiorrhiza Bunge (Danshen) 6g.	Diffuse the lung and dissolve phlegm, resolve toxins and invigorate blood.	Medicated serum	*In vitro*: respiratory syncytial virus (RSV) Long or R6 in Hep-2	20, 10, 5, 2.5, 1.25, 0.625 mg/ml	Medicated serum: 1:9 (TC_0_); 10^-3.5^/50 μl (TCID_50_)	Ribavirin	Relieves the symptoms of fever, cough and panting, regulates the Treg/Th17 balance, increases IL-10 cytokines and decreases IL-17 cytokines in RSV infected mice.	Wang et al., 2008; Dong et al., 2015; Wang et al., 2016
				*In vivo*: respiratory syncytial virus (RSV) Long in mice	4, 1.33 mg/ml				
			Oral liquid	Randomized controlled study: 507 cases of children virus pneumonia.	10, 20, 30 ml/day, (10 g/ml)	–			
Lonicera and Forsythia Epidemic-Clearing Capsule (Lianhua Qingwen Jiaonang) (Patent medicine)	Forsythia suspensa (Thunb.) Vahl (Lianqiao), Lonicera japonica Thunb. (Jinyinhua), Ephedra sinica Stapf (Zhimahuang), Prunus armeniaca L. (Kuxingren), Gypsum Fibrosum (Shengshigao), Isatis tinctoria L. (Banlangen), Dryopteris crassirhizoma Nakai (Mianmaguanzhong), Houttuynia cordata Thunb. (Yuxingcao), Pogostemon cablin (Blanco) Benth. (Guanghuoxiang), *Rheum officinale* Baill. (Dahuang), Rhodiola rosea L. (Hongjingtian), menthol and Glycyrrhiza uralensis Fisch. ex DC. (Gancao).	Clear the epidemic and resolve toxins, diffuse the lung and discharge heat.	Solution after capsule dissolution	*In vitro*: influenza virus H1N1 (FM1, PR8, Jiangxixiushui, B10, B59), RSV, parainfluenza virus (Xiantai) in A549	10, 5.55, 2.94, 1.52 g/L	FM1: 1.12 g/L (IC_50_); PR8: 1.12 g/L (IC_50_); Jiangxixiushui: 1.12 g/L (IC_50_); Brisbane-10: 1.12 g/L (IC_50_); Brisbane-59: 1.12 g/L (IC_50_); RSV: 0.50 g/L (IC_50_); Xiantai: 0.28 g/L (IC_50_)	Ribavirin	Inhibits the activity of influenza virus H1N1, parainfluenza virus, RSV, MERS- CoV or SARS-CoV *in vitro* as well as influenza A virus (H1N1) or MERS- CoV infection *in vivo*, suppresses virus-induced NF-κB activation, alleviates virus-induced gene expression of IL-6, IL-8, TNF-a, IP-10, and MCP-1, and also inhibits SARS-CoV-2 replication in Vero E6 cells and markedly reduced pro-inflammatory cytokines (TNF-α, IL-6, CCL-2/MCP-1 and CXCL-10/IP-10) production at the mRNA levels.	Zhu et al., 2003; Ding et al., 2017; Guan et al., 2018; Bao et al., 2019; Gao et al., 2020; Li R. F. et al., 2020; Xiao et al., 2020
				*In vitro*: Middle East respiratory syndrome coronavirus (MERS-CoV) in Vero cells	600, 900, 1200, 1500, 1800, 2100, 2500 μg/ml	8472 μg/ml (IC_50_)	Arbidol		
				*In vitro*: novel coronavirus (SARS-CoV-2) in Vero E6 cells	150, 300, 600 μg/ml	411.2 μg/ml (IC_50_)	Remdesivir		
				Randomized controlled study: patients with COVID-19.(Registration number of clinical trials: ChiCTR2000029601).	0.3 g/kg	–	Oseltamivir and arbidol	Relieves clinical symptoms, reduces utilization rate of anti-infective drugs, and improves patient prognosis.	
Formula I for SARS During Hyperpyrexia Peroid (Feidian Gaoreqi Yihao Fang)	Ephedra sinica Stapf (Mahuang) 5g, Prunus armeniaca L. (Xingren) 12g, Gypsum Fibrosum (Shengshigao) 45g, Anemarrhena asphodeloides Bunge (Zhimu) 10g, Lonicera japonica Thunb. (Jinyinhua) 15g, Forsythia suspensa (Thunb.) Vahl (Lianqiao) 12g, Gardenia jasminoides J.Ellis (Zhizi) 12g, Scutellaria baicalensis Georgi (Huangqin) 12g, Perilla frutescens (L.) Britton (Zisuye) 10g, Artemisia capillaris Thunb. (Yinchen) 15g, Pueraria montana var. lobata (Willd.) Maesen & S.M.Almeida ex Sanjappa & Predeep (Gegen) 15g and Pseudostellaria heterophylla (Miq.) Pax (Taizishen) 15g.	Clear heat and resolve toxins, scatter wind and diffuse the lung.	Decoction	Parallel controlled study: patients with severe acute respiratory syndrome (SARS)	0.593 g/ml, 300 ml/day	–	Ribavirin	Improves the total number of white blood cells and lymphocyte absolute value and the time of absorption of patchy shadow on chest X-ray.	Zhang R. L. et al., 2003
Formula II for SARS During Panting Peroid (Feidian Kechuanqi Erhao Fang)	Panax quinquefolius L. (Xiyangshen) 15g, Ophiopogon japonicus (Thunb.) Ker Gawl. (Maidong) 10g, Schisandra chinensis (Turcz.) Baill. (Wuweizi) 10g, Cornus officinalis Siebold & Zucc. (Shanzhuyu) 12g, Lepidium apetalum Willd. (Tinglizi) 15g, Aster tataricus L.f. (Ziwan) 15g, Eriobotrya japonica (Thunb.) Lindl. (Pipaye) 12g, Pheretima (Dilong) 12g, Salvia miltiorrhiza Bunge (Danshen) 12g, Paeonia anomala subsp. veitchii (Lynch) D.Y.Hong & K.Y.Pan (Chishao) 12g, Trollius chinensis Bunge (Jinlianhua) 8g, Scutellaria baicalensis Georgi (Huangqin) 10g and Trichosanthes kirilowii Maxim. (Gualoupi) 15g.	Clear heat and invigorate blood, boost qi and nourish yin, relieve cough and calm panting.	Decoction	Parallel controlled study: patients with severe acute respiratory syndrome (SARS)	0.527 g/ml, 300 ml/day	–	Ribavirin	Improves the total number of white blood cells and lymphocyte absolute value and the time of absorption of patchy shadow on chest X-ray.	Zhang R. L. et al., 2003
Formula III for SARS During Convalescence (Feidian Huifuqi Sanhao Fang)	Pseudostellaria heterophylla (Miq.) Pax (Taizishen) 15g, Ophiopogon japonicus (Thunb.) Ker Gawl. (Maidong) 15g, Atractylodes macrocephala Koidz. (Baizhu) 15g, Eriobotrya japonica (Thunb.) Lindl. (Zhipipaye) 15g, Wurfbainia villosa var. xanthioides (Wall. ex Baker) Skornick. & A.D.Poulsen (Sharen) 6g, Hordeum vulgare L. (Jiaomaiya) 15g, Crataegus pinnatifida Bunge (Jiaoshanzha) 15g, Astragalus mongholicus Bunge (Shenghuangqi) 15g, Pueraria montana var. lobata (Willd.) Maesen & S.M.Almeida ex Sanjappa & Predeep (Gegen) 15g, Salvia miltiorrhiza Bunge (Danshen) 15g, Citrus aurantium L. (Chenpi) 6g and Polygonatum cyrtonema Hua (Huangjing) 15g.	Boost qi and nourish yin, fortify the spleen and harmonize the stomach.	Decoction	Parallel controlled study: patients with severe acute respiratory syndrome (SARS)	0.59 g/ml, 300 ml/day	–	Ribavirin	Promotes the recovery of immune function and improves the lung inflammatory damage, improves clinical symptoms, reduces hormone dosage and shortens the course of treatment.	Zhang R. L. et al., 2003
SARS-Formula-I (Feidian Yihao Fang)	Gypsum Fibrosum (Shengshigao) 45g, Bupleurum chinense DC. (Chaihu) 15g, Anemarrhena asphodeloides Bunge (Zhimu) 10g, Fritillaria thunbergii Miq. (Zhebeimu) 10g, Scutellaria baicalensis Georgi (Huangqin) 15g, Artemisia annua L. (Qinghao) 15g, Paeonia suffruticosa Andrews (Mudanpi) 10g, Paeonia anomala subsp. veitchii (Lynch) D.Y.Hong & K.Y.Pan (Chishao) 12g, Forsythia suspensa (Thunb.) Vahl (Lianqiao) 15g, Cornus officinalis Siebold & Zucc. (Shanzhuyu) 30g, Atractylodes lancea (Thunb.) DC. (Cangzhu) 15g, Pogostemon cablin (Blanco) Benth. (Huoxiang) 15g, Coix lacryma-jobi var. ma-yuen (Rom.Caill.) Stapf (Yiyiren) 15g and Prunus armeniaca L. (Chaoxingren) 10g.	Clear heat and resolve toxins, dispel dampness and remove turbidity.	Decoction	Simple stratified randomized controlled study: patients with severe acute respiratory syndrome (SARS) in the early mild stage.	0.723 g/ml, 300 ml/day	–	Glucocorticoid, antibiotics, thymosin, and gamma globulin	Shortens the time of fever, slows down the symptoms of systemic poisoning caused by fever, promotes the absorption of pulmonary inflammation, and accelerates the reduction of glucocorticoid.	Zhang Y. L. et al., 2003; Zhang X. M. et al., 2003
				Prospective study: patients with severe acute respiratory syndrome (SARS) in the mild or severe stage.	0.58 g/ml, 400 ml/day		Glucocorticoid, ganciclovir, levofloxacin, Rocephin, Sulperazon, and thymosin		
SARS-Formula-II (Feidian Erhao Fang)	Scutellaria baicalensis Georgi (Huangqin) 15g, Artemisia annua L. (Qinghao) 15g, Trichosanthes kirilowii Maxim. (Gualou) 30g, Salvia miltiorrhiza Bunge (Danshen) 15g, Inula japonica Thunb. (Xuanfuhua) 10g, Curcuma aromatica Salisb. (Yujin) 10g, Acorus calamus var. angustatus Besser (Shichangpu) 10g, Dioscorea collettii var. hypoglauca (Palib.) S.J.Pei & C.T.Ting (Bixie) 12g, Faeces Bombycis (Cansha) 15g, Atractylodes lancea (Thunb.) DC. (Cangzhu) 15g, Atractylodes macrocephala Koidz. (Baizhu) 15g, Polyporus umbellatus (Pers.) Fries (Zhuling) 15g, Poria cocos (Schw.) Wolf (Fuling) 15g, Coix lacryma-jobi var. ma-yuen (Rom.Caill.) Stapf (Yiyiren) 15g, Prunus armeniaca L. (Chaoxingren) 10g, Plantago asiatica L. (Cheqianzi) 10g and Cornus officinalis Siebold & Zucc. (Shanzhuyu) 30g.	Clear and dispel damp-heat, diffuse the lung and direct counterflow downward.	Decoction	Simple stratified randomized controlled study: patients with severe acute respiratory syndrome (SARS) in the early mild stage.	0.757 g/ml, 300 ml/day	–	Glucocorticoid, antibiotics, thymosin, and gamma globulin	Shortens the average fever time, alleviates the systemic symptoms caused by fever, promotes the absorption of lung inflammation and accelerates the reduction of glucocorticoid.	Zhang Y. L. et al., 2003; Zhang X. M. et al., 2003
				Prospective study: patients with severe acute respiratory syndrome (SARS) in the mild or severe stage.	0.63 g/ml, 400 ml/day		Glucocorticoid, ganciclovir, levofloxacin, Rocephin, Sulperazon, and thymosin		
SARS-Formula-III (Feidian Sanhao Fang)	Panax quinquefolius L. (Xiyangshen) 30g, Astragalus mongholicus Bunge (Shenghuangqi) 30g, Cornus officinalis Siebold & Zucc. (Shanzhuyu) 30g, Ophiopogon japonicus (Thunb.) Ker Gawl. (Maidong) 15g, Anemarrhena asphodeloides Bunge (Zhimu) 10g, Fritillaria thunbergii Miq. (Zhebeimu) 10g, Patrinia scabiosifolia Link (Baijiangcao) 30g, Forsythia suspensa (Thunb.) Vahl (Lianqiao) 15g, Salvia miltiorrhiza Bunge (Danshen) 15g, Dioscorea collettii var. hypoglauca (Palib.) S.J.Pei & C.T.Ting (Bixie) 12g, Faeces Bombycis (Cansha) 15g, Coix lacryma-jobi var. ma-yuen (Rom.Caill.) Stapf (Yiyiren) 15g, Polyporus umbellatus (Pers.) Fries (Zhuling) 15g, Poria cocos (Schw.) Wolf (Fuling) 15g, Trichosanthes kirilowii Maxim. (Gualou) 30g and Aster tataricus L.f. (Ziwan) 15g.	Boost qi and nourish yin, dissolve phlegm and invigorate blood, drain dampness and direct turbidity downward.	Decoction	Simple stratified randomized controlled study: patients with severe acute respiratory syndrome (SARS) in the early mild stage.	0.923 g/ml, 300 ml/day	–	Glucocorticoid, antibiotics, thymosin, and gamma globulin	Shortens the average fever time, alleviates the systemic symptoms caused by fever, promotes the absorption of lung inflammation and accelerates the reduction of glucocorticoid.	Zhang Y. L. et al., 2003; Zhang X. M. et al., 2003
				Prospective study: patients with severe acute respiratory syndrome (SARS) in the mild or severe stage.	0.755 g/ml, 400 ml/day		Glucocorticoid, ganciclovir, levofloxacin, Rocephin, Sulperazon, and thymosin		
Compound Forsythia and Taraxaci Granule (Fufang Lianpu Keli) (Patent medicine)	Forsythia suspensa (Thunb.) Vahl (Lianqiao), Taraxacum mongolicum Hand.-Mazz. (Pugongying), Lonicera japonica Thunb. (Jinyinhua), Scutellaria baicalensis Georgi (Huangqin) and Isatis tinctoria L. (Banlangen).	Release the exterior with acrid-cool (medicinals), clear heat and resolve toxins.	Water solution	*In vitro*: novel coronavirus (SARS-CoV) BJ01 in Vero E6 cells	56 mg/ml	0.49 mg/ml (EC_50_)	Ribavirin	Inhibits SARS-CoV cultured in Vero-E6 cells.	Zhu et al., 2003
Six Spirits Capsule (Liushen Jiaonang) (Patent medicine)	*Calculus Bovis (Niuhuang), Moschus (Shexiang), Borneolum Syntheticum (Bingpian), Venenum Bufonis (Chansu), Margarita (Zhenzhu) and Realgar (Xionghuang).*	Clear heat and resolve toxins, reduce inflammation and relieve pain.	Triturated and prepared in dimethyl sulfoxide (DMSO)	*In vitro*: SARS-CoV-2 (MT123290.1) in Vero E6 cells	2.00, 1.00, 0.50, 0.25 μg/ml	0.6024 μg/ml (IC_50_); 4.930 μg/ml (TC_50_); 10^-6^/100 μl (TCID_50_)	Remdesivir	Inhibits SARS-CoV-2 virus infection *via* downregulating the expression of inflammatory cytokines induced virus and regulating the activity of NF-κB/MAPK signaling pathway *in vitro*.	Ma et al., 2020
Lung-clearing and Toxin-expelling Decoction (Qingfei Paidu Tang)	Ephedra sinica Stapf (Mahuang) 9g, Glycyrrhiza uralensis Fisch. ex DC. (Zhigancao) 6g, Prunus armeniaca L. (Xingren), Gypsum Fibrosum (Shengshigao) 10g (30g for fever), Cinnamomum cassia (L.) J.Presl (Guizhi) 9g, Alisma plantago-aquatica subsp. orientale (Sam.) Sam. (Zexie) 9g, Polyporus umbellatus (Pers.) Fries (Zhuling) 9g, Atractylodes macrocephala Koidz. (Baizhu) 9g, Poria cocos (Schw.) Wolf (Fuling) 15g, Bupleurum chinense DC. (Chaihu) 16g, Scutellaria baicalensis Georgi (Huangqin) 6g, Pinellia ternata (Thunb.) Makino (Jiangbanxia)9g, Aster tataricus L.f. (Ziwan) 9g, Zingiber officinale Roscoe (Shengjiang) 9g, Tussilago farfara L. (Kuandonghua) 9g, Iris domestica (L.) Goldblatt & Mabb. (Shegan) 9g, Asarum sieboldii Miq. (Xixin) 6g, Dioscorea oppositifolia L. (Shanyao) 12g, Citrus aurantium L. (Zhishi) 6g, Citrus aurantium L. (Chenpi) 6g and Pogostemon cablin (Blanco) Benth. (Huoxiang) 9g.	Dredge the sanjiao, clear lung heat and expel toxins, calm panting and relieve cough.	Decoction	Comparison before and after treatment: patients with COVID-19.	0.985 or 1.085 g/ml, 200 ml/day	>1,600 mg/kg (LD50)	–	Relieves cough, nasal congestion, runny nose, fatigue, anorexia, sore throat, diarrhea and other symptoms, and shows anti-inflammatory effects compared with those of only Western medicine in patients with mild and moderate COVID-19, and tends to mitigate the extent of multi-organ impairment.	Chen J. et al., 2020; Wang R. Q. et al., 2020; Xin et al., 2020; Zhou et al., 2020
				Clinical retrospective controlled study: patients with COVID-19.(Registration number of clinical trials: ChiCTR2000029778 and registration number of TCM clinical trial registry: ChiMCTR2000003003).	0.4925 or 0.5425 g/ml, 400 ml/day		Interferon, lopinavir, or arbidol		
Venting-releasing Epidemic-dispelling Granule (Toujie Quwen Keli)	Forsythia suspensa (Thunb.) Vahl (Lianqiao) 30g, *Pleione yunnanensis* (Rolfe) Rolfe (Shancigu) 20g, Lonicera japonica Thunb. (Jinyinhua) 15g, Scutellaria baicalensis Georgi (Huangqin) 10g, Persicaria tinctoria (Aiton) Spach (Daqingye) 10g, Bupleurum chinense DC. (Chaihu) 5g, Artemisia annua L. (Qinghao) 10g, Periostracum Cicadae (Chantui) 10g, Angelica decursiva (Miq.) Franch. & Sav. (Qianhu) 5g, Fritillaria cirrhosa D.Don (Chuanbeimu)10g, Fritillaria thunbergii Miq. (Zhebeimu) 10g, Prunus mume (Siebold) Siebold & Zucc. (Wumei) 30g, Scrophularia ningpoensis Hemsl. (Xuanshen) 10g, Astragalus mongholicus Bunge (Huangqi) 45g, Poria cocos (Schw.) Wolf (Fuling) 30g and Pseudostellaria heterophylla (Miq.) Pax (Taizishen) 15g.	Clear heat and resolve toxins, vent the exterior and scatter wind, boost qi and nourish yin.	Water solution	Randomized parallel controlled study: 65 cases of patients with COVID-19.	0.883 g/ml, 300 ml/day	–	Arbidol, moxifloxacin	Reduces the symptoms of patients with novel coronavirus pneumonia, and regulates the expression of peripheral blood inflammatory markers, decreases the level of CRP and increases the level of CD+ to antagonize novel coronavirus.	Fu X. X. et al., 2020
Lung-clearing, Pathogen-venting and Healthy-qi-reinforcing Formula (Qingfei Touxie Fuzheng Fang)	Ephedra sinica Stapf (Zhimahuang) 6g, Gypsum Fibrosum (Shengshigao) 20g, Prunus armeniaca L. (Xingren) 10g, Forsythia suspensa (Thunb.) Vahl (Lianqiao) 15g, Phragmites australis subsp. australis (Lugen) 30g, Lonicera japonica Thunb. (Jinyinhua) 30g, Coix lacryma-jobi var. ma-yuen (Rom.Caill.) Stapf (Yiyiren) 30g, Bombyx Batryticatus (Jiangcan) 10g, Periostracum Cicadae (Chantui) 10g, Reynoutria japonica Houtt. (Huzhang) 15g, Curcuma longa L. (Jianghuang) 10g, Paeonia lactiflora Pall. (Baishao) 10g, Pseudostellaria heterophylla (Miq.) Pax (Taizishen) 20g and Glycyrrhiza uralensis Fisch. ex DC. (Shenggancao) 15g.	Clear and diffuse lung heat, and reinforce healthy qi to consolidate the exterior.	Decoction	Randomized controlled study: Novel coronavirus pneumonia patients	0.77 g/ml; 300 400 ml/day	–	Interferon-α	Relieves the clinical symptoms of fever, cough and expectoration, chest tightness and shortness of breath, promotes the absorption of lung lesions and improves oxygenation, and reduces the content of CRP, ESR and IL-6, and increases the expression of IFN-γ to antagonize novel coronavirus.	Ding et al., 2020

*IC_50_, 50% inhibiting concentration; EC_50_, 50% maximal effective concentration; TCID_50_, 50% tissue culture infectious dose; TC_50_, median toxic concentration. TC_0_, Maximum nontoxic concentration; LD_50_, median lethal dose; LD_0_, lethal dose.

### Inhibition/Inactivation of Virus by TCM

The antiviral activity of TCM first manifests as the inhibition or inactivation of the virus. Studies have shown that a variety of TCM can directly inactivate or prevent the virus from adsorbing or penetrating into the cells, or induce the body to produce substances such as interferon, thereby inhibiting the replication of the virus.

#### Direct Inhibition of Viruses

Virus first attaches to membrane of host cells and then enters cells. After dissociation of virus particle, virus will employ host cells to replicate its genes and process proteins for viral assembly and release. In view of this series of processes, the use of drugs in the pre-infection stages of the virus can play a direct inhibitory effect on the virus. Studies showed by MTT method that the volatile oil from *Cinnamomum cassia* (L.) J. Presl and Cinnamic aldehyde could significantly inhibit the proliferation of influenza A virus (H1N1) in MDCK (Madin-Darby canine kidney) cells (*p*<0.05) (Liu et al., 2012). Ling Gou et al. confirmed that the medicated serum containing volatile oil from *Nepeta tenuifolia* Benth. and *Cinnamomum cassia* (L.) J. Presl also could significantly inhibit the proliferation of influenza A virus in MDCK cells (*p*<0.05) and show a certain degree of direct killing of virus (Gou et al., 2013). Studies have shown that Glycyrrhizic acid from *Glycyrrhiza uralensis* Fisch. ex DC. not only directly inhibits the replication of coronavirus, but also acts on the early stage of virus adsorption and membrane penetration, which may be related to its activation of protein kinase C, casein kinase II and nuclear transcription factor B (Cinatl et al., 2003). Arctiin and its aglucone, arctigenin from the fruits of *Arctium lappa* L. showed potent *in vitro* antiviral activities against influenza A virus (A/NWS/33, H1N1) (IFV). Based on the data from time-of-addition experiments and on release tests of progeny viruses, arctigenin was assumed to interfere the early event(s) of viral replication after viral penetration into cells, and to suppress the release of progeny viruses from the host cells (*p*<0.01 or *p*<0.001) (Hayashi et al., 2010). The classical Chinese medical formula, Pueraria Decoction (Gegen Tang) can play an antiviral role in the adsorption stage of virus (*p*<0.01), and Pueraria decoction and its antiviral activity are positively correlated with dose (Geng et al., 2019). Neuraminidase (NA) can be another target molecule for antiviral effect. Neuraminidase is a mushroom cloud tetramer glycoprotein located on the envelope of influenza virus and involved in virus release and spread. Jiawei Liu et al. used chromatographic separation technology to screen and isolate Eugeniin, an effective compound from *Syzygium aromaticum* (L.) Merr. & L.M.Perry, showing that it could inhibit neuraminidase activity of H1N1 *in vitro* (Liu et al., 2018). Studies by Kaotan Chen et al. showed that resveratrol, (E)-3,5,12-trihydroxystilbene-3-O-β-D-glucopy-ranoside-2’-(3″,4″,5″-trihydroxybenzoate) and Catechin-3-O-gallate, three extracts of *Reynoutria japonica* Houtt., could effectively inhibit neuraminidase activity (Chen et al., 2012). Baicalin is a flavonoid in *Scutellaria baicalensis* Georgi. Studies by Yue Ding et al. have confirmed that baicalin can significantly inhibit the neuraminidase activity of influenza A (H1N1) virus (*p*<0.05 or *p*<0.01) (Ding et al., 2014). Han-Bing Li et al. found that acidic sugars in Isatis tinctoria L. had higher inhibitory activity of neuraminidase than neutral sugars and total sugars. Moreover, acidic sugars were slightly stronger to inhibit activity of neuraminidase in H5N1 influenza virus than that in H1N1 virus (*p*<0.05) (Li et al., 2009). Xianying Yang et al. used UPLC-Q-TOF-MS (Ultra-high performance liquid chromatography coupled with a four-pole time-of-flight mass spectrometer) to detect the neuraminidase inhibitory activity of *Rhus chinensis* Mill., and found that ethyl acetate, ethanol extract, acyl-pentagalic glucose, ellagic acid and gallic acid from Wubeizi exhibit different levels of neuraminidase inhibitory activity (Yang et al., 2017). Studies by Shuang Lang et al. showed that the compound Iso-ginkgo biloba diflavone extracted from *Osmunda japonica* Thunb. has a significant inhibitory effect on the neuraminidase activity of influenza virus (Lang et al., 2019). Gao Chen et al. evaluated the ratio of combination of two Chinese medicines of *Coptis chinensis* Franch. and *Magnolia officinalis* Rehder & E.H. Wilson and found that the best inhibitory effect on neuraminidase was at 1:1 ratio (Chen et al., 2017). Traditional Chinese medical formulae and proprietary traditional Chinese medicine products such as Ephedra, Apricot Kernel, Gypsum and Licorice Decoction (Maxingshigan Tang), medicated serum of Folium Ginkgo and Ephedra Lung-clearing Capsule (Yinhuang Qingfei Jiaonang), as well as Heat-toxin-clearing Injection (Reduning Zhusheye) also exhibit inhibitory effect of neuraminidase (*p*<0.05 or *p*<0.01) (Hsieh et al., 2012; Sun et al., 2014; Li et al., 2015).

#### Indirect Inhibition of Viruses

Interferon is a glycoprotein produced by cells stimulated by viruses or other interferon inducers. After binding to interferon receptors, it can induce cells to produce antiviral proteins with enzyme activity, such as protein kinase and 2’,5’-adenosine kinase (Der and Lau, 1995; Min and Krug, 2006), thereby inhibiting viral replication. Studies by Rong Liu et al. showed that the volatile oil and cinnamaldehyde contained in *Cinnamomum cassia* (L.) J.Presl could increase the content of IFN-α and IFN-β in serum of mice with viral pneumonia (*p*<0.05) (Liu et al., 2013). Ting He et al. documented that *Nepeta tenuifolia* Benth. can increase the levels of IFN-α, IFN-β and IL-2 in virus-infected mice (*p*<0.05 or *p*<0.01) (He et al., 2013). Hongri Xu et al. showed that *Scutellaria baicalensis* Georgi could increase the expression of the antiviral factor IFN-γ in lung tissue (*p*<0.05) (Xu et al., 2019). Cheng-Chuan Tsou pointed out that the antiviral effect of *Arctium lappa* L. was related to its ability to induce the organism to produce interferon (*p*<0.05 or *p*<0.01) (Tsou, 2007).

### Regulatory Effects on Immune and Cellular Inflammatory Factors

Excessive immune response and release of inflammatory cytokines are important causes of viral pneumonia and lung injury. In patients infected with SARS, Influenza A (H1N1) virus and SARS-CoV-2 (Li et al., 2012; Casadevall and Pirofski, 2014; Huang et al., 2020), abnormally-elevated inflammatory cytokines so called cytokine storm can be detected and are closely related to disease severity. Therefore, inhibiting the overexpression of inflammatory factors and improving immune function have become important part in the treatment of viral pneumonia.

#### Regulation of the TLR-NF-κB Signaling Pathway

The TLR-NF-κB signaling pathway is an important pathway that mediates the expression of inflammatory factors. Toll-like receptor (TLR) is a transmembrane protein located on the cell membrane, which is composed of extracellular region, transmembrane region and intracellular region. At present, there are three kinds of TLR3, TLR7 and TLR8 which are closely related to the virus. These three receptors plus TLR9 have functional domain inside the cell, while the rest of the receptors are expressed outside the cell. Whether the different distribution of this functional domain is related to its antiviral effect needs further study. After virus invasion, both TLR3-mediated MyD88 independent signaling and TLR7-mediated MyD88-dependent signaling ultimately activate the nuclear transcription factor NF-κB, which induces and promotes the expression of preinflammatory factors (Sanjeewa et al., 2020). Yongfeng Wang et al. and Li Wang et al. established a mouse model of influenza viral pneumonia and found that gardenin from *Gardenia jasminoides* J.Ellis and baicalin from *Scutellaria baicalensis* Georgi could significantly reduce the expression of IL-6, TNF-α, TLR3 and TRIF mRNA in the lung tissue of mice (*p*<0.01) (Wang et al., 2014; Wang Y. F. et al., 2020). At the same time, studies have shown that gardenin and baicalin can also inhibit the TLR7/MyD88 pathway to reduce NF-κB activation (*p*<0.05 or *p*<0.01) (Zhang and Yu, 2010; Wan et al., 2014). Yuhuan Xie et al. found that essential oil of *Nepeta tenuifolia* Benth. can inhibit TRAF6 protein expression in the lung tissue of mice, and have a certain inhibitory effect on MyD88 to achieve anti-influenza virus pneumonia (*p*<0.05) (Xie Y. H. et al., 2007). Chinese medical formulas Sweet Wormwood and Scutellaria Gallbladder-Clearing Decoction (Haoqin Qingdan Tang), Pueraria Decoction (Gegen Tang), Wind-scatering and Lung-diffusing Formula Granule (Shufeng Xuanfei Fang Keli), Exterior-releasing and Interior-clearing Formula Granule (Jiebiao Qingli Fang Keli), and Lonicera, Forsythia, Bupleurum and Cinnamon Twig Formula II (Yinqiaochaigui Erhao Fang) all could inhibited expression of TLR7, MyD88, NF-κB and decreased serum TNF-α, IL-1 and IL-6 levels (*p*<0.05 or *p*<0.01) (Lai et al., 2011; Liu et al., 2014; Li et al., 2018; Geng et al., 2019).

#### Regulation of the PI3K/Akt Signaling Pathway

The PI3K/Akt signaling pathway can also activate the nuclear transcription factor NF-κB, which induces the expression of inflammatory factors (Harikrishnan et al., 2018). Studies have shown that resveratrol [from *Morus alba* L. (Sangshen), *Reynoutria japonica* Houtt. (Huzhang), *Veratrum nigrum* L. (Lilu) or *Senna tora* (L.) Roxb. (Juemingzi)] can inhibit the expression of PI3K and NF-κB in the lung tissues of infected mice (*p*<0.05) (Li M. et al., 2020). Xiaoxue Liu showed that polysaccharides and flavonoids from *Morus alba* L. could significantly reduce the expressions of PI3K, AKT1/2 and NF-κBp65, as well as IL-4 and INF-γ in serum of respiratory syncytial virus (RSV)-infected mice (*p*<0.05) (Liu, 2016). Xiaoxue Liu et al. also pointed out that Lung-clearing and Collaterals-unblocking Ointment (Qingfei Tongluo Gao) applied on the back of mice could also inhibit the expression of PI3K and NF-κB proteins in lung tissue induced by respiratory syncytial virus, thereby reducing inflammation and protecting lung tissue (*p*<0.05) (Liu X. X. et al., 2016). Shuling Nan et al. found that Ascending and Descending Powder (Shengjiang San) can reduce the excessive expression of NF-κB protein in the lung tissue, significantly improve the content of lung sIgA, IL-10, IL-IRα and sTNFR, reduce the serum IL-1β, IL-6, TNF-α content, inhibit proinflammatory factor and induce suppression of inflammatory factor expression to reduce pulmonary inflammatory injury (*p*<0.05 or *p*<0.01) (Nan et al., 2016a; Nan et al., 2016b). In addition, researchers found that hypericin and hyperoside extracted from *Hypericum perforatum* L. could reduce the expression of IL-6 and TNF-α in lung tissue and serum of mice infected with influenza A virus, and increase the expression of IFN-γ and IL-10 protein (*p*<0.05 or *p*<0.01) (Wang et al., 2009). Wei Luo et al. found that the levels of TNF-α and IL-10 in serum and lung tissues of mice infected with influenza virus were reduced by electroacupuncture and moxibustion at bilateral Feishu (BL 13) on the back of mice (*p*<0.01) (Luo et al., 2014).

#### Regulation of Lymphocyte Subsets

As one of the three lines of defense, cellular immunity plays an important role in eliminating pathogens. Experimental studies have shown that *Andrographis paniculata* (Burm.f.) Nees and *Ilex asprella* (Hook. et Arn.) Champ. ex Benth. can increase the percentage of CD3+ lymphocytes in the T-lymphocyte subsets in peripheral blood of mice infected with influenza virus, and regulate the CD4/CD8 ratio to enhance the immune function of mice (Chen et al., 2016; Wang et al., 2019). *Hypericum perforatum* L. extract can improve the immunologic function of influenza virus-infected mice by enhancing T and B lymphocyte conversion, phagocytic function of macrophages and NK killing activity (*p*<0.05 or *p*<0.01) (Xu et al., 2016). Gegen decoction can regulate the ratio of CD3+CD4+/CD3+CD8+ and CD4+IFN-γ+/CD4+IL-4+ in peripheral blood of virus-infected mice (*p*<0.01) (Geng et al., 2019). Shengjiang Power can increase the percentage of CD8+ in peripheral blood, regulate the radio of CD4+/CD8+, and improve the immune function of the body (*p*<0.05 or *p*<0.01) (Nan et al., 2016b). Other study results have shown that compared with ribavirin, the Haoqin Qingdan Decoction can improve the ratio of T lymphocyte subgroup and Th1/Th2 cell balance more effectively in rats with damp-heat syndrome of influenza viral pneumonia (Zhang et al., 2013).

### TCM Protecting Host Cells

Some traditional Chinese medicines have been studied, which do not directly inhibit virus replication or regulate immune and inflammatory factors, but protect host cells and increase their tolerance to viruses. Shanshan Guo et al. found that the extract ZG from *Gardenia jasminoides* J. Ellis can improve the host cell membrane fluidity after infection of parainfluenza virus type 1 (PIV-1) (*p*<0.01) and maintain its normal function therefore to play an antiviral role (Guo et al., 2007).

### Clinical Research

Compared with single herbs, traditional Chinese medical formulas are more widely used in the clinical prevention and treatment of viral pneumonia. Studies have shown that proprietary traditional Chinese medicine product or Chinese medical formula decoction plays a certain role in anti-inflammatory, immune regulation, inhibition of viral replication, prevention of viral cytopathic disease and improvement of pathology (See **Table 2**).

### TCM for the Treatment of SARS Coronavirus Pneumonia

Pneumonia caused by SARS coronavirus is a highly infectious pneumonia that can involve multiple organ lesions. The main clinical manifestations are fever, cough, headache, fatigue, aching pain of muscle and joint, oppression in chest, and dyspnea, etc. Tietao Deng, a master of Chinese medicine, considered that it belongs to the category of spring epidemic and damp-heat pestilence diseases, which pathogeneses are accumulation of damp-heat toxin, consumption of Qi and damage of Yin easily, and existance of blood stasis. According to Tietao Deng, SARS can be divided into early, middle, extreme and recovery stages (Deng, 2003). In the process of clinical treatment, therapeutic outcomes of combined treatment with TCM and western medicine are usually better than that of Western medicine alone in terms of release of clinical symptoms, improvements of pneumonia and blood oxygen saturation as well as the count of lymphocyte and T cell subsets. For example, Ruilin Zhang et al. treated 49 SARS patients with integrated traditional Chinese and western medicine. Beside the basic treatment, patients were respectively given Formula I for SARS during Hyperpyrexia Peroid (Feidian Gaoreqi Yihao Fang) with high fever to clear heat and resolve toxins, scatter wind and diffuse the lung, Formula II for SARS during Panting Peroid (Feidian Kechuanqi Erhao Fang) with cough and panting to clear heat and invigorate blood, boost qi and nourish yin, relieve cough and calm panting, Formula III for SARS during Convalescence (Feidian Huifuqi Sanhao Fang) with recovery to boost qi and nourish yin, fortify the spleen and harmonize the stomach. The results showed that the remission time of clinical symptoms and reduced hormone usage in the integrated TCM western medicine group were 2.52 days and 222.69 mg respectively, shorter than those in the control group, and the difference was statistically significant difference (*p*<0.05). Moreover, TCM also played an important role in promoting the recovery of immune function and reducing pulmonary inflammatory injury (Zhang R. L. et al., 2003). Yunling Zhang et al. employed integrated TCM and western medicine to treat 65 SARS patients, prescribing SARS-Formula-I (Feidian Yihao Fang) at the high fever stage to clear heat and resolve toxins, dispel dampness and remove turbidity; SARS-Formula-II (Feidian Erhao Fang) at panting and oppression stage to clear and dissolve damp-heat, diffuse the lung and direct counterflow downward; and SARS-Formula-III (Feidian Sanhao Fang) at the absorbing stage to boost qi and nourish yin, dissolve phlegm and invigorate blood, drain dampness and direct turbidity downward, respectively. The results showed that the treatment of integrated traditional Chinese and western medicine had advantages over the western medicine alone in terms of reducing fever, relieving clinical symptoms, absorbing pulmonary inflammatory lesions and reducing hormone usage (*p*<0.001 or *p*<0.05) (Zhang Y. L. et al., 2003). Jianping Liu et al. performed meta-analysis on the treatment of SARS with integrated Chinese and western medicine, and found that the combined Chinese and western medicine treatment could shorten the clinical symptoms and fever time, reduce secondary fungal infection, and relieve pulmonary inflammation (Liu et al., 2005).

### TCM for the Treatment of Influenza Virus Pneumonia

Influenza virus pneumonia is a common pulmonary infection disease in clinic. Its symptoms often see fever, cough, bitter taste in the mouth, dry throat, throat pain, even visible high fever, heavy panting, profuse sweating, etc. Shouchuan Wang et al. used Lung-clearing Oral Liquid (Qingfei Koufuye) with effects of diffusing the lung and dissolving phlegm, resolving toxins and invigorating blood to treat infantile viral pneumonia with a pattern of phlegm-heat blocking the lung. The results showed the efficacy of Lung-clearing Oral Liquid was better than ribavirin injection in term of reducing fever, cough, asthma and inflammation (*p*<0.05 or *p*<0.01) (Wang et al., 2016). Based on the conventional treatment, Youzhong Tian et al. gave Two Roots Lung-clearing Beverage (Ergen Qingfei Yin) and Phlegm-heat-clearing Injection (Tanreqing Zhusheye) to treat patients with A (H1N1) viral pneumonia. The results showed that compared with the western medicine control group, combining Chinese and western medicine treatment could significantly reduce the content of serum inflammatory cytokines, such as TNF-alpha, IL-6, IL-8 and c-reactive protein, and the antipyretic and antitussive effect was better than that of the control group (*p*<0.01) (Tian et al., 2019). Fengmei Sang et al. used Sweet Wormwood and Scutellaria Gallbladder-Clearing Decoction (Haoqin Qingdan Tang) to treat patients with virus pneumonia with a pattern of damp-heat for a week as the observation group; the level of CD3+ and CD4+ was significantly higher than that of the control group patients (receiving conventional treatment); and the level of NF-κB was significantly lower than that of the control group patients (*p*<0.05). The total effective rate of the observation group was higher than that of the control group, with statistical significance (*p*<0.05) (Sang et al., 2014).

### TCM for the Treatment of Coronavirus Induced Disease 2019 (COVID-19)

Coronavirus induced disease 2019 is a novel coronavirus pneumonia and characterized by fever, dry cough and fatigue as the main symptoms, accompanied by nasal congestion, runny nose, sore throat, muscle soreness and pain, etc. In severe cases, breathing difficulties and hypoxemia will occur, or patients develop into acute respiratory distress syndrome, septic shock, uncorrectable metabolic acidosis, coagulation dysfunction, and multi-organ failure and so on (General Office of National Health Commission of the People’s Republic of China and Office of National Administration of TCM, 2020). TCM classifies COVID-19 as “epidemic disease”. Raoqiong Wang et al. applied Lung-clearing and Toxin-expelling Decoction (Qingfei Paidu Tang) to treat 98 patients with COVID-19 and found that Lung-clearing and Toxin-expelling Decoction could significantly improve the liver and kidney functions of patients such as ALT and AST, recover the D-dimer, plasma C-reactive protein and erythrocyte precipitation, significantly reduce fever, cough (dry cough), asthma, pharyngeal pain, fatigue, anorexia and other symptoms, as well as relieve adverse reactions of antiviral drugs (*p*<0.01) (Wang R. Q. et al., 2020). Compared to 36 COVID-19 patients treated with oral abidor tablets and ambroxol tablets as the control group, Xiaoxia Fu et al. applied Venting-releasing Epidemic-dispelling Granule (Toujie Quwen Keli) to 37 COVID-19 patients as the treatment group. The results showed that compared with the western medicine treated control group, the combination of Chinese and western medicine treatment group can increase the absolute lymphocyte value and decrease C-reactive protein. CD4+ count and CD4+/CD8+ ratio were better than those in the control group (p<0.05) (Fu X. X. et al., 2020). Yunfei Qu et al. used the modified Ephedra, Apricot Kernel, Gypsum and Licorice Decoction (Maxingshigan Tang) [*Ephedra sinica* Stapf (Mahuang), *Prunus armeniaca* L. (Xingren), *Gypsum Fibrosum* (Shengshigao), *Platycodon grandiflorus* (Jacq.) A. DC. (Jiegeng), *Eriobotrya japonica* (Thunb.) Lindl. (Pipaye), ** *Atractylodes macrocephala* Koidz. (Baizhu), *Poria cocos* (Schw.) Wolf (Fuling), *Fritillaria cirrhosa* D.Don (Chuanbeimu), *Scutellaria baicalensis* Georgi (Huangqin), *Morus alba* L. (Sangbaipi) and *Glycyrrhiza uralensis* Fisch. ex DC. (Zhigancao)] with conventional western medicine to treat 40 patients with ordinary COVID-19, and found that after three days of treatment, IL-6 level significantly decreased compared with that before the treatment (*p*<0.05), and levels of AST, ALT and creatinine were normal. After 7 days of treatment, IL-6 level decreased to normal, hypersensitive C-reactive protein level decreased significantly, CD4+T and CD8+T cell count increased significantly compared with that before treatment (*p*<0.05), levels of AST, ALT and creatinine were still normal. The results showed that this modified decoction had a significant effect on common COVID-19 without significant hepatorenal toxicity (Qu Y. F. et al., 2020). Ming Liu et al. evaluated the combination of traditional Chinese and western medicine for treatment of COVID-19. Based on the treatment with Lung-clearing, Pathogen-venting and Healthy-qi-reinforcing Formula (Qingfei Touxie Fuzheng Fang), Wind-scattering Toxins-resolving Capsule (Shufeng Jiedu Jiaonang), and Lonicera and Forsythia Epidemic-Clearing Granule (Lianhua Qingwen Keli) and so on respectively, the combination of traditional Chinese medicine and western medicine had better outcome than western medicine alone in several clinical aspects such as reducing severe conversion rate, shortening hospitalization time and improving the patients’ clinical symptoms such as fever, cough, fatigue and oppression in chest (Liu et al., 2020). Furthermore, there are many other proprietary traditional Chinese medicine products also play an important therapeutic role by direct antiviral actions, antipyretic and analgesic, immune-regulation, anti-inflammation, and anti-acute lung injury, such as Agastache Qi-Correcting Capsule (Huoxiang Zhengqi Jiaonang), Lonicera Fever-clearing Granule (Jinhua Qinggan Keli), Wind-scattering Toxin-resolving Capsule (Shufeng Jiedu Jiaonang), Xiyanping Injection (Xiyanping Zhusheye), Blood-clearing Injection (Xuebijing Zhusheye), Ginseng and Aconite Injection (Shenfu Zhusheye), Ginseng and Ophiopogon Injection (Shenmai Zhusheye), Peaceful Palace Bovine Bezoar Pill (Angong Niuhuang Wan), etc. (Zhuang et al., 2020).

## Discussion

In the treatment of viral pneumonia through syndrome differentiation, TCM plays a variety of roles in inhibiting the proliferation, replication, adsorption and membrane penetration of the virus, promoting the expression of interferon *in vivo*, inhibiting inflammatory reaction, enhancing immunity, etc., which is one of the theoretical bases for the clinical application of TCM in the prevention and treatment of viral pneumonia. Viruses with RNA genetic material, such as influenza virus and coronavirus, are more likely to mismatch and cause mutations in the replication process than DNA viruses (Woolhouse et al., 2016). Their high variability makes it more difficult to develop vaccines and more susceptible to drug resistance to single chemical drugs. Traditional Chinese herbal medicine and compound medicinals are characterized by multi-component, multi-pathway and multi-pathway complex networks. Therefore, drug resistance is relatively rare in the clinical practice of TCM. Moreover, in the process of diagnosis and treatment of TCM, treatment based on differentiation of symptoms and signs, especially treatment based on classification of symptoms and signs, can best reflect the overall concept of TCM. TCM has precise therapeutic activity and less adverse reactions. Accumulating evidence has demonstrated the competent therapeutic effects of TCM against viral pneumonia with a prominent safety profile. TCM has obvious characteristics and great advantages on syndrome differentiation for the prevention and treatment of viral pneumonia before specific antiviral drugs and vaccines are developed and produced. However, TCM in treatment of viral pneumonia still have some problems. First, theoretical study of viral pneumonia in TCM, especially regarding the pathogenesis and changes of the virus are not comprehensive, systematic and in-depth; second, the complexity of traditional Chinese herbal medicine composition and its compound makes it difficult to understand mechanism and action and less specific; third, in the process of treatment of viral pneumonia, it is usually carried out by traditional and macroscopic methods, with strong subjectivity, which cannot be considered as microscopic and specific as modern medical diagnostic standards. There is still a lack of recognized and unified standards for the classification of TCM Syndrome of viral pneumonia; fourth, the basic research on prevention and treatment of SARS-CoV-2 and COVID-19 with TCM is less developed, which may be related to that case collection of infectious disease in different countries is different or may not be allowed, and/or lack of laboratories that meet the requirements for conducting research of contagious diseases. Furthermore, the TCM treatment was mainly based on decoction, which makes difficult to set up control group therefore, generate greater varieties.

The basic treatment of medical formula and proprietary traditional Chinese medicine product are mainly based on the analysis of the whole process of the etiology and pathogenesis of viral pneumonia, which include grasping the basic pathogenesis, establishing the basic treatment method, and combining the viewpoint of modern medicine, formulating the basic prescriptions, or adding or subtracting along with the syndromes, or further changing the dosage form, and developing the treatment method for patent medicine. Although this kind of treatment often lacks the concept of TCM syndrome and the flexibility of syndrome differentiation and treatment, it has been proved to be effective in practice due to its grasp of the basic pathogenesis of the disease and the application of various methods. Moreover, it has been reported successful many times and seems to become a distinct alternative choice alongside the classical approach. Furthermore, in addition to oral administration of TCM decoction or pills, intravenous administration of TCM and other methods have been reported as another treatment of viral pneumonia. In addition, there are also many reports about the external treatment of patients with viral pneumonia, such as the atomizing inhalation of traditional Chinese medicine extract, external application of Chinese medicine powder or paste, foot reflexology, infantile massage, etc., all which reveal some new ideas and ways for TCM treatment of viral pneumonia.

In recent years, researches on viral pneumonia by TCM mainly focus on influenza virus, mainly on mice or cell models infected by influenza A (H1N1) virus, while researches on SARS-CoV and MERS-CoV are few. Studies on such viruses as SARS-CoV, MERS-COV, H1N1 and other viruses should be conducted in P3 laboratory (biosafety level 3 laboratory) or higher biosafety laboratory. Extensive and in-depth studies on the prevention and treatment of viral diseases with TCM are subject to certain conditions. Fortunately, in 2020, the Ministry of Science and Technology of China issued the “Guidance on Strengthening the Biosafety Management of Novel Coronavirus High-level Virus Microbiology Laboratory”, requiring the laboratory to play a role as a platform to serve the needs of scientific and technological research. This will provide strong policy support for the in-depth study of the antiviral effect and mechanism of TCM. Although the research on the antiviral activity of TCM has been performed with molecular biology, the specific therapeutic effects of traditional Chinese herbal medicine or compound on virus and pneumonia remains to be further investigated because of its complex components. Therefore, in order to better treat viral pneumonia with TCM based on syndrome differentiation and the overall concept of theoretical system, we should adhere to the theory of TCM as the basis, actively combine with modern or western medicine, complement each other, and use modern science and technology to explore the role and mechanism of viral pneumonia and traditional Chinese medicine in a more comprehensive, systematic and in-depth way, deeply analyze the characteristics of viral pneumonia syndromes, unify evidence pattern classification standards, further standardize and unify the evaluation criteria of syndrome differentiation and efficacy in order to facilitate the communication of clinical and scientific research work, use new diagnostic techniques to prevent misdiagnosis and missed diagnosis, and establish positive drug control in a standardized way in the process of clinical research to improve the credibility of TCM treatment. In the future, the TCM treatment theory and clinical application of viral pneumonia should pay special attention to strengthen experimental research, especially the effective Chinese medicine compounds. The precise mechanism of Chinese medicine in the treatment of viral pneumonia should be scientifically clarified to achieve the synchronization of clinical research and experimental research. In this way, Chinese medicine can be better to treat patients with viral pneumonia in a scientific and standardized manner based on syndrome differentiation.

## Conclusions

TCM has been widely used in basic and clinical researches of virus diseases especially viral pneumonia in human. Some Chinese medicine has shown certain therapeutic effect, but high-quality experimental design and randomized clinical controlled study are still needed. A wide variety of antiviral traditional Chinese herbal medicines also provides potential opportunity for further development in specific therapeutic agents to treat viral pneumonia around the world.

## Author Contributions

YL and SX wrote the manuscript. YL and LY helped in searching for related articles. Y’aY, LQ, TL, and SX proofread the manuscript. SX and YG guided the writing and critically revised the manuscript. All authors contributed to the article and approved the submitted version.

## Funding

This work was supported by the Fundamental Research Funds for the Central Universities of China (No.20720200012), Beijing University of Chinese Medicine Research Project (No.2020-JYB-YJ-004) (Emergent project for the prevention and control of coronavirus pneumonia) and the Top Young Scientist Funds and Top Young Doctor Funds of Beijing University of Chinese Medicine (No.BUCM-2019-JCRC007 and BUCM-2019-QNMYB011).

## Conflict of Interest

The authors declare that the research was conducted in the absence of any commercial or financial relationships that could be construed as a potential conflict of interest.
